# Physiology-guided beat-level arrhythmia classification from ECG using a CNN-transformer hybrid neural network

**DOI:** 10.3389/fcvm.2026.1802210

**Published:** 2026-07-08

**Authors:** Guangfeng Li, Zhidong Zhang, Gang Qiao, Xiaosan Chen, Gangqiang Zhou, Kavimbi Chipusu

**Affiliations:** 1 Grand Vascular Surgery, Heart Center of Henan Provincial People’s Hospital, Central China Fuwai Hospital, Central China Fuwai Hospital of Zhengzhou University, Zhengzhou, China; 2 Department of Mechanical Engineering, Division of Biomedical Engineering, University of Saskatchewan, Saskatoon, SK, Canada

**Keywords:** AAMI heartbeat categories, arrhythmia classification, CNN–Transformer hybrid, electrocardiogram (ECG), gated fusion, multi-head self-attention

## Abstract

**Background:**

Accurate ECG-based arrhythmia classification is essential for large-scale screening and continuous monitoring, but recognition remains challenging because diagnostic cues are distributed across local waveform morphology and temporal rhythm context.

**Methods:**

We developed TransECG-Net, a physiology-guided CNN–Transformer hybrid network for AAMI-aligned five-class heartbeat classification. The CNN branch extracts local P–QRS–T morphology, QRS-width variation, and amplitude-shape features, while the Transformer branch models global temporal dependencies using positional encoding and multi-head self-attention. Both representations are combined through a learnable dimension-wise gated fusion module. Public ECG recordings were segmented into fixed-length heartbeat windows and split into training, validation, and testing subsets using a stratified 70%/15%/15% protocol. Performance was assessed using accuracy, precision, recall, specificity, and F1-score, with additional noise-robustness and edge-device evaluation.

**Results:**

TransECG-Net correctly classified 4,976 of 5,000 testing samples, achieving 99.52% accuracy. Class-wise F1-scores were 99.90% for N, 99.43% for L, 99.56% for R, 99.61% for A, and 99.12% for V, with a macro-averaged F1-score of 99.52%. It outperformed DeepECG-Net (98.30%) and Hybrid CNN-BLSTM (94.20%) while maintaining 35 ms latency and 28 MB memory footprint.

**Conclusion:**

TransECG-Net supports accurate, physiology-guided, noise-tolerant, and edge-deployable ECG arrhythmia screening for wearable and clinical monitoring.

## 1 Introduction

### 1.1 Research background and significance

Cardiovascular diseases and rhythm-related disorders remain a leading cause of morbidity and mortality worldwide (1, 2). Cardiac arrhythmias are clinically critical because they may evolve from transient rhythm disturbances into severe adverse outcomes, including thromboembolism, syncope, heart failure exacerbation, and sudden cardiac arrest. Electrocardiography (ECG) is a cornerstone for arrhythmia screening and diagnosis, providing a non-invasive and cost-effective measure of cardiac electrical activity that is suitable for both point-of-care assessment and long-term monitoring. In routine practice, clinicians interpret ECG recordings by analyzing waveform morphology and temporal relationships—such as the P wave, QRS complex, T wave, and associated intervals—to determine rhythm status. However, manual ECG interpretation is time-consuming and highly dependent on experience, which limits scalability in high-throughput screening and continuous monitoring, particularly with the rapid growth of wearable devices and telehealth services that generate large volumes of ECG data.

Beyond ECG-only screening, automated cardiovascular assessment also benefits from broader biomedical signal, imaging, and hemodynamic modeling. ECG signal analysis has been combined with statistical clinical indicators in cardiopulmonary assessment (3), while cardiovascular flow and morphology have been investigated through hemodynamic simulations, cardiac-flow reconstruction, stenosis-related vascular modeling, and cardiac image segmentation for structural assessment (4–8). These studies further support the need for AI frameworks that consider physiological structure, signal behavior, and clinically interpretable cardiovascular evidence together.

In real-world applications, the primary demand extends beyond achieving high accuracy on curated datasets to delivering reliable, real-time screening under diverse physiological conditions and environmental disturbances. To align with clinical screening workflows, this study focuses on the AAMI-recommended five-class heartbeat classification scheme, which supports practical triage by enabling automated front-end filtering of predominantly normal beats while highlighting suspicious patterns for further clinical confirmation. Such a triage-oriented formulation is consistent with clinical practice: automated systems can efficiently reduce the review burden by prioritizing potentially abnormal events—such as ectopic beats, supraventricular abnormalities, and bundle-branch-block–related patterns—thereby improving early detection efficiency and supporting timely intervention.

Despite its clinical relevance, developing a robust and deployable heartbeat classifier remains challenging. First, ambulatory and wearable ECG signals are frequently contaminated by motion artifacts, baseline wander, muscle noise, and electrode-contact disturbances, often resulting in low signal-to-noise ratios that can substantially degrade model performance if robustness is not explicitly considered. Second, practical deployment scenarios impose strict constraints on latency, memory footprint, and computational cost. For bedside monitoring and wearable devices, models should provide low-latency inference with limited hardware resources, making edge-friendly architectures preferable to purely cloud-dependent solutions. Therefore, ECG intelligence for real-world screening must simultaneously balance discriminative capacity, noise robustness, and computational efficiency.

Beyond performance and deployment constraints, ECG modeling also faces privacy and data governance barriers. Centralized training with large-scale multi-institution data is frequently restricted by regulatory requirements and institutional policies that limit raw patient data sharing. Federated learning provides a promising alternative by enabling collaborative model optimization while keeping data locally stored. However, ECG data distributions are often non-identically distributed (non-IID) across institutions, populations, and device types, which can impair generalization and training stability in federated settings. Consequently, there is a strong need for an arrhythmia classification framework that can maintain performance under heterogeneous data and real-world noise while remaining efficient for on-device inference.

Motivated by these considerations, the goal of this work is to develop an ECG arrhythmia classification framework that simultaneously achieves: (1) strong discriminative performance for AAMI five-class heartbeat recognition under both clean and noisy conditions, (2) real-time inference with low resource consumption suitable for edge deployment, and (3) privacy-preserving training capability under federated learning with heterogeneous, non-IID data. Addressing these requirements provides a scalable foundation for AI-assisted ECG screening systems that can support continuous monitoring, reduce clinician workload, and enhance the reliability of early abnormality detection in both clinical and home-care environments.

### 1.2 Detection and classification of cardiovascular diseases based on traditional machine learning

Traditional machine learning (ML) methods have long played an important role in intelligent ECG analysis and remain widely adopted in practical screening pipelines. In classical workflows, ECG signals are typically processed through denoising, baseline correction, and normalization, followed by segmentation into individual beats or fixed-length windows. Handcrafted features are then extracted to capture clinically meaningful patterns, including time-domain descriptors (e.g., RR intervals and heart rate variability indices), morphological characteristics (e.g., QRS width, P–R/Q–T intervals, and amplitude statistics), and frequency-domain or time–frequency representations (e.g., spectral power and wavelet coefficients). These engineered features are subsequently fed into conventional classifiers such as Support Vector Machines (SVM), K-Nearest Neighbor (KNN), Logistic Regression (LR), Decision Trees (DT), Random Forests (RF), extreme learning machines, and gradient-boosting models to perform arrhythmia detection or multi-class classification (9–18).

A primary advantage of traditional ML lies in its relatively low computational demand and its compatibility with low-dimensional, structured representations, making it attractive for resource-constrained settings and small-sample scenarios. Moreover, certain models (e.g., DT/RF) provide partial interpretability via rule-based structures or feature importance analysis, which can assist in clinical understanding and algorithm debugging. Therefore, when robust feature engineering is available, traditional ML pipelines can provide a lightweight and deployable solution for early-stage ECG screening tasks. More broadly, hybrid representation-learning and classical predictor combinations, such as autoencoder–extreme learning machine designs, have also been used for nonlinear time-series prediction, illustrating the value of compact learned representations in resource-aware predictive modeling (19).

However, real-world ECG monitoring, particularly in wearable and telehealth contexts, exposes several fundamental limitations of traditional ML approaches. First, feature extraction and classification are decoupled, and overall performance is highly dependent on handcrafted descriptors; subtle morphological variations and context-dependent rhythm patterns may not be sufficiently represented. Second, ambulatory ECG is frequently contaminated by motion artifacts, baseline wander, muscle noise, and electrode-contact disturbances, which can cause feature drift and degrade robustness across varying signal-to-noise ratios. Third, generalization across heterogeneous devices, acquisition protocols, and patient populations is often limited, making it difficult to maintain consistent performance in large-scale deployments. These challenges are amplified under the AAMI five-class heartbeat classification setting, where the system must reliably distinguish diverse abnormal beats that may not conform to a single fixed template.

To address these issues, deep learning has increasingly become the mainstream paradigm for ECG intelligence by enabling end-to-end representation learning from raw or minimally processed ECG signals (20–27). Convolutional Neural Networks (CNNs) can automatically learn discriminative local morphological patterns (e.g., QRS-related structures) and multi-scale features, thereby reducing reliance on manual feature engineering. Nevertheless, ECG is inherently sequential, and modeling temporal dependency is essential for capturing rhythm dynamics and contextual information beyond local waveform shapes. Accordingly, Recurrent Neural Networks (RNNs), especially Long Short-Term Memory (LSTM), bidirectional LSTM, and Gated Recurrent Unit (GRU) architectures, have been widely explored to integrate temporal context over ECG sequences or windows (28–31). LSTM-based sequence modeling has also been applied in other dynamic-system prediction and control problems, highlighting the wider utility of recurrent models for temporally dependent signals (32). While these recurrent models can strengthen sequence modeling, their step-wise computation may limit parallel efficiency and can increase inference latency in certain deployment settings.

More recently, attention mechanisms and Transformer-based encoders have demonstrated strong capability in capturing long-range dependencies and global context via self-attention, which is beneficial for rhythm-level characterization and context-aware ECG interpretation. In parallel, recent ECG deep learning studies have increasingly emphasized the importance of jointly learning morphology-related and temporal discriminative information. For example, Han et al. proposed MTDL-NET, a morphological and temporal discriminative learning framework for heartbeat classification, demonstrating that waveform morphology and temporal representation provide complementary evidence for distinguishing heartbeat categories (33). This dual-feature philosophy is highly consistent with clinical ECG interpretation, where local waveform changes such as QRS morphology, amplitude variation, and beat-shape deformation must be interpreted together with temporal rhythm context and beat-to-beat dependency. Building on this progress, hybrid architectures that combine CNN-based local feature extraction with Transformer-based global dependency modeling have emerged as an effective design strategy: CNN modules emphasize fine-grained morphology, whereas self-attention captures broader temporal relationships across the entire segment. This hybrid paradigm is particularly suitable for practical ECG screening, as it supports data-driven feature learning under noisy conditions and can be engineered to meet edge-device constraints through controlled model depth, embedding dimension, and attention configuration.

Motivated by the above developments, this study adopts a hybrid CNN–Transformer framework for AAMI five-class heartbeat classification, with the aim of achieving robust performance under noisy conditions and enabling efficient real-time inference on edge hardware. To ensure fair evaluation and continuity with prior ECG sequence-modeling literature, we include representative recurrent baselines (e.g., CNN–LSTM and CNN–GRU/CNN–BiLSTM, depending on availability) alongside CNN-only and attention-based alternatives, enabling a systematic assessment of the benefits introduced by the proposed hybrid attention-driven design under a unified dataset setting and evaluation protocol.

### 1.3 Cardiovascular disease diagnostics using TransECG-Net hybrid architecture

To address the practical requirements of large-scale screening and continuous ECG monitoring, we propose TransECG-Net, a hybrid deep-learning architecture for real-time arrhythmia classification under heterogeneous and noisy acquisition conditions. Different from traditional pipelines that depend on handcrafted features and separate classifiers, and also beyond purely recurrent sequence models, TransECG-Net performs end-to-end representation learning by integrating convolutional morphology extraction with a Transformer encoder for global context modeling. This design aims to jointly capture local waveform characteristics and long-range temporal dependencies, which are both essential for clinically meaningful ECG interpretation. Compared with prior morphology–temporal learning frameworks such as MTDL-NET (33), TransECG-Net follows the same physiological motivation but implements it through a CNN-based morphology branch, a Transformer-based temporal-context branch, and a learnable dimension-wise gated fusion module that adaptively balances the two complementary feature sources for each ECG segment.

Technically, TransECG-Net first employs multi-scale 1D convolutional blocks to learn discriminative morphological representations from raw ECG segments, emphasizing clinically relevant structures such as P–QRS–T complexes, QRS-duration variations, and amplitude/shape irregularities. The extracted local descriptors are then fed into a Transformer encoder, where positional encoding and multi-head self-attention enable the model to capture global temporal relationships across the entire window. Compared with recurrent computation, the self-attention formulation offers improved parallelism and a controllable modeling capacity for long-range dependencies, which is advantageous when designing for low-latency inference under practical deployment constraints.

In the target setting, TransECG-Net outputs a five-class probability distribution over the AAMI heartbeat categories (N, L, R, A, V). To align with clinical screening workflows, we additionally incorporate a triage-oriented decision strategy: the model rapidly identifies normal beats/segments, while non-normal predictions are aggregated to flag suspicious segments for prioritized clinician review. This configuration supports efficient front-end screening in continuous monitoring scenarios, reducing unnecessary manual review while maintaining sensitivity to potentially abnormal events.

Beyond classification accuracy, real-world ECG systems must remain stable under noise contamination and device variability. Accordingly, we evaluate TransECG-Net under controlled SNR perturbations to assess robustness to motion artifacts, baseline wander, and electrode-contact noise that are common in wearable and ambulatory recordings. Moreover, to support clinical feasibility outside centralized computing environments, we validate the model for edge deployment, demonstrating that strong predictive performance can be achieved with a constrained memory footprint and low inference latency on lightweight hardware platforms. Such deployment readiness is critical for continuous monitoring, timely alerting, and practical integration into home-care and remote-health services.

From a broader healthcare systems perspective, the growing demand for ECG screening and follow-up monitoring continues to outpace limited cardiology resources. Automated ECG intelligence can alleviate clinician workload by enabling scalable preliminary screening and streamlining diagnostic workflows, especially in high-throughput outpatient settings and telemedicine. Related healthcare prediction studies have also shown the usefulness of ensemble learning when direct measurements are limited or when scalable clinical decision support is needed (34). However, high-performing models typically benefit from multi-institutional data, while raw medical data sharing is constrained by privacy regulations and governance policies. To address this barrier, we further extend TransECG-Net within a federated learning framework, enabling collaborative training across distributed clients while keeping patient data local. This privacy-preserving paradigm supports scalable model improvement under realistic non-IID data distributions and provides a pathway toward clinically deployable ECG intelligence that balances accuracy, robustness, efficiency, and privacy.

Accordingly, the physiology-guided nature of TransECG-Net is reflected in three linked design choices: beat-centered ECG input preparation, morphology-oriented convolutional feature extraction, and temporal-context modeling through Transformer self-attention, followed by adaptive gated fusion of these complementary physiological representations. Finally, to ensure a fair and reproducible evaluation, we include representative baselines commonly used in ECG sequence modeling—such as CNN-only and CNN–RNN variants (e.g., CNN–LSTM/CNN–GRU/CNN–BiLSTM) and attention-based alternatives—under the same dataset and protocol, enabling a direct assessment of the performance and deployment advantages introduced by the proposed TransECG-Net design.

## 2 Materials and methods

In this study, we develop an automated real-time ECG arrhythmia classification framework based on TransECG-Net, a hybrid deep-learning model that couples a CNN-based morphology extractor with a Transformer encoder for global context modeling. The overall pipeline consists of three components: (i) ECG data preparation and annotation under an AAMI-aligned five-class heartbeat setting, (ii) end-to-end model training and inference using the proposed CNN–Transformer architecture, and (iii) comprehensive evaluation under noise perturbations, edge-device deployment constraints, and privacy-preserving federated learning scenarios.

The study utilizes publicly available ECG resources, including the MIT-BIH Arrhythmia Database and the PhysioNet/CinC 2017 dataset, to reflect both canonical arrhythmia recordings and diverse real-world acquisition conditions. Heartbeat-level labels are organized into a practical five-class formulation consistent with the AAMI recommendation (N, L, R, A, V). (If an additional label mapping or beat extraction procedure is applied to non-beat–annotated records, the corresponding segmentation and mapping rules are specified to ensure reproducibility.).

Raw ECG recordings are standardized through filtering/baseline correction and amplitude normalization, and then segmented into fixed-length windows (or beat-centered segments) to generate uniform input samples for model training and real-time inference. This processing produces consistent signal length and scale across subjects and devices, facilitating stable optimization and deployment.

Given an input ECG segment, the CNN backbone first extracts local morphological representations associated with clinically meaningful waveform patterns (e.g., P–QRS–T complexes and QRS-duration–related changes), producing compact feature maps that emphasize shape and amplitude characteristics relevant to arrhythmia discrimination. These features are then passed to a Transformer encoder that performs global sequence modeling using multi-head self-attention, enabling the network to capture long-range temporal dependencies across the entire segment without recurrent gating operations. Because self-attention alone does not inherently encode temporal order, positional encoding is incorporated to preserve sequential structure. Finally, a softmax-based classifier outputs class probabilities over the AAMI-aligned five heartbeat categories.

To assess robustness under realistic ambulatory conditions, controlled noise perturbations are injected into clean ECG windows after segmentation. Following commonly used signal corruption protocols, Gaussian white noise and baseline wander artifacts are added to simulate motion artifacts, electrode-contact disturbance, and drift commonly observed in wearable monitoring. Signal-to-noise ratio (SNR) levels are varied from 0 to 20 dB, and the noisy inputs are evaluated under the same inference settings to quantify performance degradation and robustness.

For real-world deployability, TransECG-Net is evaluated on an embedded edge platform (Raspberry Pi 4B, quad-core ARM Cortex-A72, 1.5 GHz, 4 GB RAM). Practical real-time metrics—including inference throughput, end-to-end latency, and memory footprint—are reported to characterize feasibility for continuous monitoring scenarios.

To support privacy-preserving learning across distributed environments, TransECG-Net is further assessed under a federated learning configuration in which multiple clients train locally and only model updates are communicated to a central server. Global aggregation is performed using FedAvg across five simulated clients under non-IID data distributions, reflecting realistic heterogeneity in normal/abnormal proportions across sites and devices. This setting enables evaluation of training stability and generalization under privacy and governance constraints.

Model performance is quantified using standard classification metrics (accuracy, precision, recall, F1-score, and class-wise measures where appropriate). The proposed method is compared against representative baselines—including CNN-only models and sequence-modeling alternatives (e.g., LSTM/GRU-based variants) as well as established ECG deep-learning architectures—under the same dataset split and evaluation protocol to isolate the benefit of attention-based global dependency learning for real-time arrhythmia classification.

### 2.1 Datasets and evaluation protocol

This study used publicly available ECG recordings from PhysioNet resources to construct an AAMI-aligned five-class heartbeat classification dataset (35). The primary beat-level annotations were obtained from the MIT-BIH Arrhythmia Database, and ECG recordings from the PhysioNet/CinC 2017 dataset were used to enrich signal variability and noise-related diversity during preprocessing and robustness evaluation. All ECG signals were standardized using the same preprocessing pipeline, including denoising, amplitude normalization, and segmentation into fixed-length heartbeat windows. Each eligible heartbeat window was assigned to one of five clinically interpretable categories: normal beat (N), left bundle branch block beat (L), right bundle branch block beat (R), atrial premature beat (A), and ventricular premature beat (V).

The dataset was divided into training, validation, and testing subsets using a stratified 70%/15%/15% split at the heartbeat-sample level. Accordingly, the reported results should be interpreted as an intra-patient beat-level classification evaluation, in which heartbeat samples from the same subject may appear across different subsets. We note that inter-patient splitting is a more stringent evaluation protocol because it completely separates subjects between training and testing, thereby avoiding subject overlap (12, 13). Nevertheless, for consistency and fair benchmarking, the same heartbeat-level stratified split protocol was applied to all compared models throughout this study.

To improve transparency and reproducibility, the class distribution used for model development is summarized in Table 1. The testing distribution was kept consistent with the confusion-matrix evaluation reported in the Results section.

**Table 1:** Distribution of AAMI five-class ECG heartbeat samples used for model development.

Class	Clinical meaning	Training, *n*	Validation, *n*	Testing, *n*	Total, *n*
N	Normal beat	4,578	981	981	6,540
L	Left bundle branch block beat	4,513	967	967	6,447
R	Right bundle branch block beat	4,769	1,022	1,022	6,813
A	Atrial premature beat	4,732	1,014	1,014	6,760
V	Ventricular premature beat	4,741	1,016	1,016	6,773
Total	—	23,333	5,000	5,000	33,333

The table summarizes the number of ECG heartbeat samples assigned to each AAMI-aligned class across the training, validation, and testing subsets. The five classes include normal beat (N), left bundle branch block beat (L), right bundle branch block beat (R), atrial premature beat (A), and ventricular premature beat (V). The dataset was divided using a stratified 70%/15%/15% protocol, yielding 23,333 training samples, 5,000 validation samples, and 5,000 testing samples. The class distribution table improves transparency regarding sample balance and supports reproducibility of the evaluation protocol.

The final dataset contained 33,333 heartbeat samples, including 6,540 N beats, 6,447 L beats, 6,813 R beats, 6,760 A beats, and 6,773 V beats. The samples were divided into training, validation, and testing subsets using a stratified 70%/15%/15% partition, yielding 23,333 training samples, 5,000 validation samples, and 5,000 testing samples. The testing distribution was kept consistent with the confusion-matrix evaluation reported in the Results section.

Class imbalance was addressed during both data partitioning and model training. First, stratified splitting was used to preserve the relative distribution of the five heartbeat categories across the training, validation, and testing subsets. Second, minority-class compensation was applied during training using class-balanced sampling and weighted cross-entropy loss. For each class *k*, the class weight was computed inversely proportional to its frequency in the training set is given in Equation (1):

$$w_{k}=\frac{N}{Kn_{k}}$$ (1)

where *N* is the total number of training samples, *C* is the number of heartbeat classes, and *n_c* is the number of training samples belonging to class c. The weighted cross-entropy loss was then defined in Equation (2) as:

$$L=-\frac{1}{B}\overset{B}{\underset{i=1}{\sum }}\;\overset{K}{\underset{k=1}{\sum }}\;w_{k}y_{ik}\log({\hat{y}}_{ik})$$ (2)

where *B* is the batch size, *y_ic* is the ground-truth label indicator for sample *i* and class *c*, and *p_ic* is the predicted probability for class *c*. This imbalance-aware training strategy reduces bias toward relatively frequent classes and improves sensitivity to less frequent abnormal heartbeat categories. Importantly, no resampling was applied to the validation or testing subsets; therefore, the final evaluation reflects the original held-out class distribution.

### 2.2 Pre-processing

ECG recordings acquired in practical monitoring scenarios are often affected by motion artifacts, baseline wander, and other non-stationary disturbances, which may obscure diagnostically relevant waveform characteristics. To ensure consistent model inputs for real-time arrhythmia classification, all ECG signals from the two public datasets were standardized through a unified preprocessing pipeline. Specifically, the raw ECG recordings were first segmented into fixed-length windows, after which noise artifacts were removed and the signal amplitude was normalized to reduce variability introduced by motion artifacts and inter-record differences. This preprocessing generates uniform ECG windows suitable for training and inference in the proposed five-class classification setting (five heartbeat categories). Related ECG preprocessing studies have used R-peak detection, baseline-wander removal, empirical-mode decomposition, wavelet-domain denoising, variational decomposition, and morphological filtering to improve signal quality before classification (36–42).

To keep the normalization step explicit and reproducible, we apply min–max scaling to map each ECG window into the range [0,1] is given in Equation (3) as follows:.

$$x_{\mathrm{norm}}=\frac{x-X_{\min}}{X_{\max}-X_{\min}}$$ (3)

where *x* denotes the original ECG sample value within a window, and $X_{\min}$ and $X_{\max}$ are the minimum and maximum values in that window, respectively.

In addition, to evaluate robustness under realistic ambulatory noise conditions, controlled noise perturbations were introduced after segmentation following PhysioNet ECG corruption guidelines. In particular, Gaussian white noise and baseline wander artifacts (based on MIT-BIH Noise Stress Test Database specifications) were added to clean ECG windows at SNR levels from 0 to 20 dB, implemented using MATLAB's awgn() function. This procedure simulates common wearable monitoring disturbances (e.g., motion artifacts and intermittent disconnections) and enables systematic assessment of noise resilience.

### 2.3 Physiology-guided model design

TransECG-Net was designed to reflect the way clinicians interpret ECG signals, where arrhythmia recognition depends on both local waveform morphology and temporal rhythm context. In this study, the term “physiology-guided” does not refer to manually engineered diagnostic rules, but to an architecture and input strategy that explicitly align model components with physiologically meaningful ECG evidence.

First, each ECG segment was centered on heartbeat-level information so that the model could learn local morphology associated with the P wave, QRS complex, ST–T segment, and surrounding baseline. This design allows the convolutional branch to focus on short-duration morphological changes such as QRS widening, abnormal ventricular depolarization patterns, altered beat shape, and amplitude variations. These features are particularly relevant for distinguishing bundle branch block beats and ventricular premature beats from normal beats.

Second, the Transformer branch was introduced to model temporal dependencies across the full ECG window. Although local morphology is important, some arrhythmia-related patterns require broader contextual interpretation, including beat-to-beat dependency, premature timing, rhythm irregularity, and the temporal relationship between adjacent waveform components. The positional encoding and multi-head self-attention mechanism allow the model to preserve sequential order and assign higher attention to diagnostically informative time regions within the ECG segment.

Third, the five output categories were organized according to an AAMI-aligned heartbeat classification setting: normal beats, left bundle branch block beats, right bundle branch block beats, atrial premature beats, and ventricular premature beats. This grouping links the model output directly to clinically interpretable electrophysiological patterns rather than arbitrary data-driven clusters. Therefore, the classification task was structured around ECG categories that correspond to recognizable conduction or ectopic-beat mechanisms.

Finally, physiology-guided learning was implemented through gated integration of morphology-dominant and context-dominant representations. The CNN branch provides local waveform evidence, whereas the Transformer branch provides global temporal evidence. The gated fusion module adaptively adjusts the relative contribution of these two sources for each input segment. For example, beats with strong local QRS abnormalities may rely more heavily on CNN-derived morphology, while beats requiring timing or contextual interpretation may receive greater contribution from Transformer-derived temporal features. In this way, the proposed framework preserves the clinical logic of ECG interpretation while retaining end-to-end feature learning.

### 2.4 Convolutional neural network

Convolutional Neural Networks (CNNs) are widely used for representation learning from 1D biomedical time-series signals such as ECG, where local waveform morphology provides critical evidence for abnormality screening. In TransECG-Net, the CNN module serves as the first-stage feature extractor, aiming to reduce the dimensionality of raw ECG signals while learning localized discriminative patterns closely related to clinically meaningful structures (e.g., P–QRS–T/PQRST waves).

Specifically, the input ECG window $X_{\mathrm{input}}\in R^{n\times 1}$ is convolved with learnable 1D kernels $W\in R^{k\times 1}$ and bias *b*, producing a feature map $X_{\mathrm{feature}}$ that encodes local morphological characteristics. The convolution operation can be expressed in Equation (4) as:

$$X_{\mathrm{feature}}=\mathrm{Conv}(X_{\mathrm{input}},W,b)$$ (4)

The resulting feature maps contain informative representations of both normal and abnormal rhythm patterns, forming a compact and noise-tolerant basis for downstream modeling. Batch normalization was used after convolutional layers to stabilize training and improve convergence during end-to-end optimization (43). After CNN-based feature extraction, these representations are passed to the Transformer encoder, which further captures long-range temporal dependencies across the ECG window for arrhythmia classification.

### 2.5 Transformer branch and dimension-wise gated fusion

The Transformer branch was used to model temporal dependencies that extend beyond local waveform morphology. For each ECG window, the single-channel input sequence was first projected into a 128-dimensional embedding space. Sinusoidal positional encoding was then added to preserve the sequential order of ECG samples before self-attention was applied. The embedded sequence was processed by two Transformer encoder layers, each consisting of multi-head self-attention, a position-wise feed-forward network, residual connections, layer normalization, and dropout. The output sequence was summarized by adaptive average pooling to generate a fixed-length temporal-context feature vector, denoted as $t\in R^{128}$, as shown in Equation (5).

$$t\in R^{128}$$ (5)

In parallel, the CNN branch produced a morphology feature vector, denoted as $c\in R^{256}$, after residual 1D convolutional blocks and adaptive average pooling. Because the CNN and Transformer branches generate features with different dimensions, both representations were first projected into the same latent feature space of dimension H=256:.

$$\hat{c}=W_{c}c+b_{c},\hat{t}=W_{t}t+b_{t}$$ (6)

In Equation (6), $\hat{c}\in R^{256}$ and $\hat{t}\in R^{256}$ denote the projected CNN-derived and Transformer-derived features, respectively. The two projected vectors were then concatenated and passed through a learnable gating network, which is given in Equation (7):

$$g=\sigma (W_{2}\delta (W_{1}[\hat{c};\hat{t}]+b_{1})+b_{2})$$ (7)

where $\left[\hat{c};\hat{t}\right]$ denotes feature concatenation, δ(⋅) denotes the ReLU activation, σ(⋅) denotes the sigmoid function, and $g\in R^{256}$ is a dimension-wise gate. Each element of *g* ranges from 0 to 1 and determines the relative contribution of the CNN and Transformer features at the corresponding latent dimension.

The final fused representation was computed in Equation (8) as:

$$f=g⊙\hat{c}+(1-g)⊙\hat{t}$$ (8)

where ⊙ denotes element-wise multiplication. Therefore, the fusion module does not simply concatenate or average the two branches. Instead, it learns an adaptive feature-level balance between local morphology and temporal context. When a latent dimension is more informative for waveform morphology, the corresponding gate value increases the contribution of the CNN-derived feature. Conversely, when rhythm context or long-range temporal dependency is more informative, the gate assigns greater weight to the Transformer-derived feature. The fused feature vector $f\in R^{256}$ was then passed through a fully connected layer, ReLU activation, dropout, and the final softmax classifier to generate five-class heartbeat probabilities.

### 2.6 Structure of CNN–Transformer model

**Figure 1:**
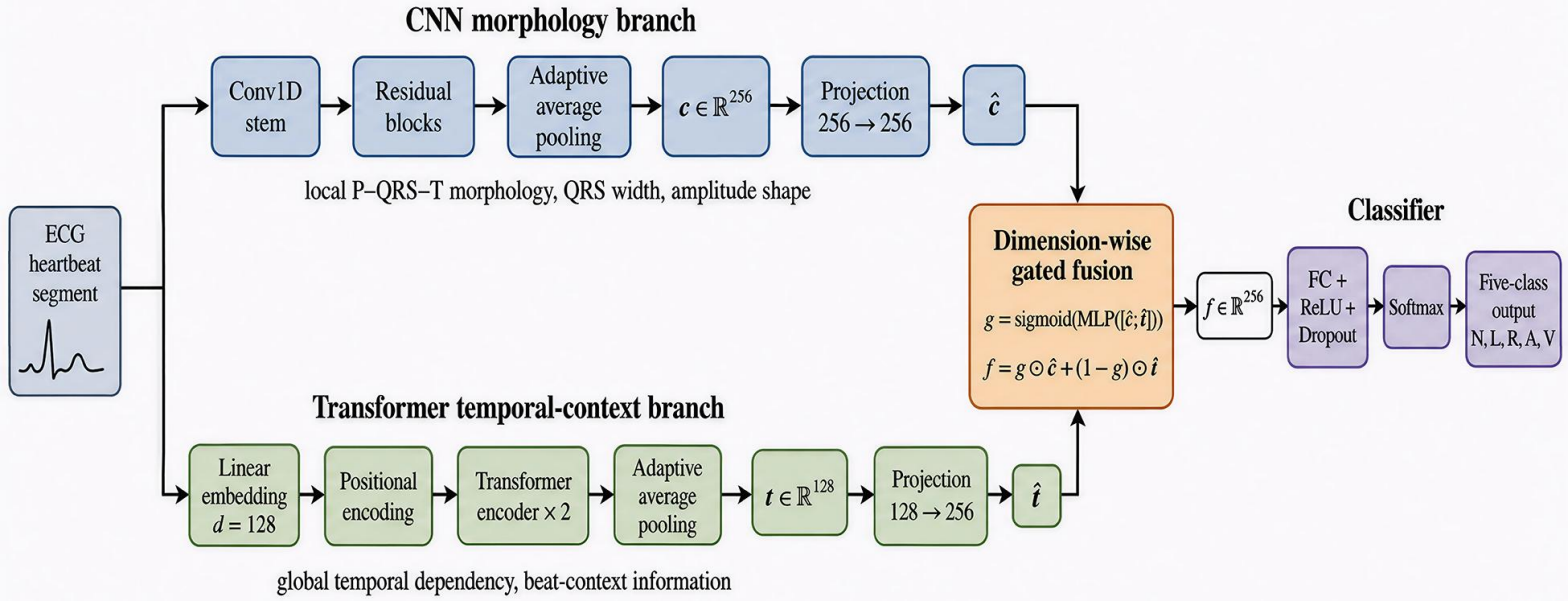
Architecture of TransECG-Net for AAMI five-class ECG heartbeat classification. The normalized ECG heartbeat segment is processed through two parallel feature-learning branches. The CNN morphology branch extracts local waveform features related to P–QRS–T morphology, QRS-width variation, and amplitude-shape changes, producing a morphology-sensitive representation. In parallel, the Transformer temporal-context branch embeds the same ECG segment and applies positional encoding with multi-head self-attention to model broader temporal dependencies and beat-context information. The CNN-derived and Transformer-derived features are projected into a shared latent space and integrated using a dimension-wise gated fusion module, which adaptively balances morphology-dominant and context-dominant evidence. The fused representation is then passed through fully connected layers and a softmax classifier to generate the final AAMI-aligned five-class output: N, L, R, A, and V.

The proposed CNN–Transformer architecture comprises three key components: (i) a CNN branch for extracting local morphological patterns, (ii) a Transformer branch for modeling long-range temporal dependencies, and (iii) a learnable gated fusion module that adaptively integrates the two representations for arrhythmia classification. As illustrated in Figure 1, the model follows a parallel two-branch design. The CNN branch receives the normalized heartbeat segment and extracts local morphology-sensitive representations through stacked residual 1D convolutional blocks. These features mainly encode short-range waveform evidence, including P–QRS–T morphology, QRS-width variation, local amplitude change, and beat-shape deformation. In parallel, the Transformer branch embeds the same ECG window into a 128-dimensional sequence representation and applies positional encoding and multi-head self-attention to capture temporal context across the full segment. After adaptive pooling, the CNN feature vector $c\in R^{256}$ and Transformer feature vector $t\in R^{128}$ are projected into a shared 256-dimensional latent space. A dimension-wise gating network then generates $g\in R^{256}$, which adaptively controls the relative contribution of the two branches according to $f=g⊙\hat{c}+(1-g)⊙\hat{t}$. This design makes the fusion stage input-dependent rather than fixed, allowing TransECG-Net to emphasize morphology-dominant evidence for conduction-related abnormalities and context-dominant evidence for rhythm-dependent or premature-beat patterns. Detailed module configuration and dimensional specifications are provided in Table 2.

**Table 2:** Overall pipeline and module-wise specification of the proposed TransECG-Net framework.

Component	Description
Input	Heartbeat segments of length L = 188 samples (single-lead ECG).
Stem	Conv1D(1→64), kernel=7, stride=1, padding=3 + BatchNorm+ReLU.
CNN branch: ResidualBlock1	2 × Conv1D(64→64), kernel=3, stride=1.
CNN branch: ResidualBlock2	2 × Conv1D(64→128), kernel=3, stride=2.
CNN branch: ResidualBlock3	2 × Conv1D(128→256), kernel=3, stride=2.
CNN branch: AdaptiveAvgPooling	Pool over time dimension → c ∈$R^{256}$.
Transformer branch: LinearEmbedding	Input [B,L,1] → [B,L,128].
Transformer branch: PositionalEncoding	Sinusoidal positional encoding, d = 128.
Transformer branch: TransformerLayer×2	MHSA (nhead=4) + FFN (256) + dropout=0.1 → [B,L,128].
Transformer branch: AdaptiveAvgPooling	Pool over sequence length → t ∈$R^{128}$.
Fusion: ProjectionLayers	CNN: 256→256; Transformer: 128→256 (shared latent H = 256).
Fusion: GatingNetwork	MLP(512→256→256) + sigmoid → g ∈$R^{256}$.
Fusion: Fused feature	f = g ⊙ c∼ + (1−g) ⊙ t∼.
Classifier	FC(256→128) + ReLU+dropout=0.3; Output FC(128→5) + Softmax.

The table presents the architectural components of TransECG-Net from input ECG segment to final five-class classification output. It summarizes the single-lead heartbeat input length, convolutional stem, residual CNN blocks, Transformer embedding and encoder layers, adaptive pooling operations, projection layers, dimension-wise gated fusion module, and final classifier. The table clarifies how local morphology-sensitive CNN features and temporal-context Transformer features are generated, dimensionally aligned, fused, and converted into class probabilities for N, L, R, A, and V heartbeat categories.

The complete TransECG-Net architecture, including the parallel CNN morphology branch, Transformer temporal-context branch, projection layers, dimension-wise gated fusion module, and final classifier, is illustrated in Figure 1.

**Figure 2:**
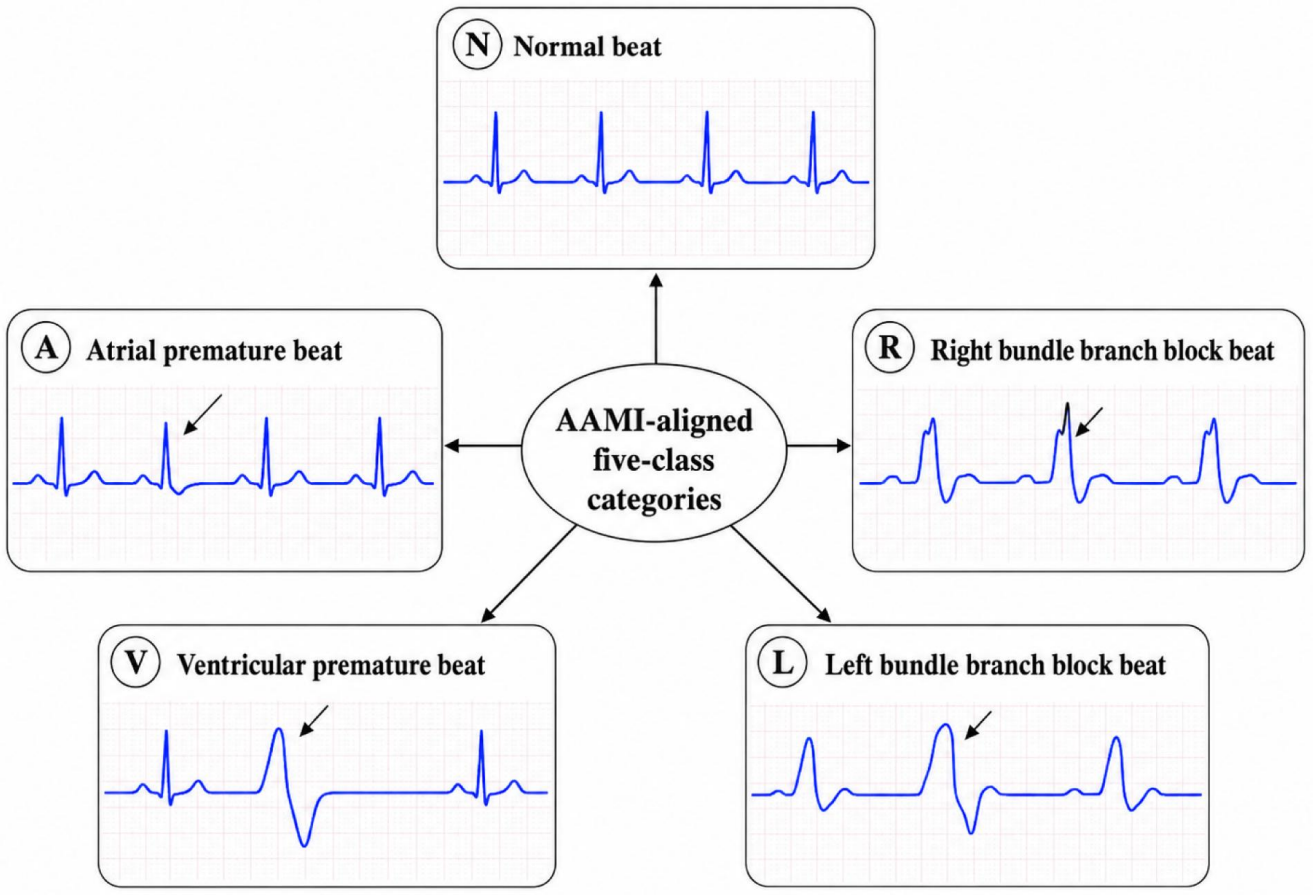
Representative ECG waveform examples of the five heartbeat categories used for classification. Representative single-lead ECG waveform examples are shown for the study's AAMI-aligned five-class heartbeat setting: normal beat (N), left bundle branch block beat (L), right bundle branch block beat (R), atrial premature beat (A), and ventricular premature beat (V). The figure illustrates clinically interpretable morphology and timing differences, including QRS-shape alteration, bundle-branch-block–related conduction patterns, and premature ectopic beats. These waveform characteristics provide the physiological basis for the CNN morphology branch and support the study's physiology-guided classification design.

After defining the network architecture, representative ECG waveforms were used to clarify the physiological meaning of the five heartbeat categories learned by the model. Representative single-lead ECG waveform examples for the five AAMI-aligned heartbeat categories are shown in Figure 2.

These waveform categories form the physiological basis of the classification task, while the corresponding model components used to learn morphology and temporal context are summarized in Table 2.

With the architectural pipeline defined, the experimental settings used for training, robustness testing, federated learning, and edge-device evaluation were then specified.

### 2.7 Experimental setting

The experiments were designed to evaluate TransECG-Net under (i) standard supervised training, (ii) noise-robustness stress testing, (iii) privacy-preserving federated learning, and (iv) edge-device real-time feasibility. Model training was conducted on a high-performance setup using an NVIDIA RTX 3060 GPU (16 GB RAM), while real-time inference and deployment-oriented benchmarking were performed on a Raspberry Pi 4B (1.5 GHz Quad-core ARM Cortex-A72, 4 GB RAM) to reflect resource-constrained wearable/edge conditions.

For model development, the ECG dataset was split into 70% training, 15% validation, and 15% testing, with each fixed-length ECG window labeled under a five-class classification setting (five heartbeat categories) for scalable screening.

To quantify robustness under ambulatory noise, Gaussian white noise and baseline wander artifacts were injected into clean ECG segments across SNR levels from 0 to 20 dB, following PhysioNet signal corruption guidelines; noise was added post-segmentation using MATLAB awgn().Performance was reported both under noise-free vs. noisy conditions and across SNR levels (0–20 dB).

To support distributed training without sharing raw ECG data, a federated learning simulation was conducted with five client nodes; local model updates were aggregated on a central server using FedAvg, including evaluation under non-IID client data distributions, with only minimal performance degradation reported in heterogeneous settings.

Finally, edge feasibility was validated on Raspberry Pi, reporting practical deployment metrics including latency (<50 ms), throughput (∼800 signals/s), and battery/runtime behavior (≈ 15% battery consumption over 12 h with a 5 V 5000 mAh LiPo), demonstrating suitability for real-time monitoring and wearable use cases. The dataset preparation, preprocessing, evaluation, federated-learning, and edge-deployment settings are summarized in Table 3.

**Table 3:** Dataset preparation, preprocessing, evaluation settings, and deployment configuration.

Category	Setting	Value/Description
Training platform	GPU workstation	NVIDIA RTX 3060 GPU, 16 GB RAM
Edge evaluation platform	Embedded device	Raspberry Pi 4B, 1.5 GHz Quad-core Cortex-A72, 4 GB RAM
Task formulation	Labels	Five-class: N, L, R, A, V (AAMI heartbeat categories)
Data split	Train/Val/Test	70%/15%/15%
Noise robustness	Noise types	Gaussian white noise+baseline wander artifacts
Noise robustness	SNR levels	0–20 dB (PhysioNet guidelines; MATLAB awgn())
Federated learning	Clients	5 client nodes (simulation)
Federated learning	Aggregation	FedAvg at server
Federated learning	Data heterogeneity	non-IID client distributions; <∼1.3% degradation reported
Edge metrics	Real-time inference	∼800 signals/s; latency <50 ms
Edge metrics	Energy test	5 V 5,000 mAh LiPo; ∼15% battery used over 12 h

The table summarizes the experimental settings used to train, validate, and test TransECG-Net. It includes the task formulation, data split, noise robustness protocol, federated learning configuration, training platform, edge evaluation platform, and deployment metrics. Noise robustness was assessed using Gaussian white noise and baseline wander artifacts across SNR levels from 0 to 20 dB. Edge feasibility was evaluated using a Raspberry Pi 4B platform, with inference latency, throughput, and memory footprint reported to assess real-time monitoring suitability.

These settings were used consistently across the proposed model and baseline comparisons, while the detailed model hyperparameters are provided separately in Table 4.

**Table 4:** Model architecture and training hyperparameters of TransECG-Net.

Module	Parameter	Value	Notes/Rationale
Input	Window length/sampling	L = 188 samples (single-lead ECG)	Consistent with the segmentation setting used throughout the manuscript.
Embedding (Transformer)	Embedding dimension	d = 128	Linear embedding followed by positional encoding.
Embedding (Transformer)	Positional encoding	Sinusoidal (sin/cos)	Preserves temporal order for self-attention.
Transformer encoder	Number of encoder layers	2	As implemented in TransECG-Net.
Transformer encoder	Number of attention heads	nhead=4	MHSA configuration used in the Transformer encoder.
Transformer encoder	FFN hidden dimension	256	Feed-forward sublayer width.
Regularization	Dropout	Transformer: 0.1; Classifier: 0.3	Keep consistent with architecture table.
Transformer pooling	Sequence aggregation	Adaptive average pooling over length → t ∈$R^{128}$	Produces fixed-length global representation.
CNN branch → Fusion	CNN feature dimension (after pooling)	c ∈$R^{256}$	CNN branch outputs 256-d vector before fusion.
Fusion	Projection layers	CNN: 256→256; Transformer: 128→256 (latent H = 256)	Aligns modalities for gated fusion.
Fusion	Gating network	MLP(512→256→256) + sigmoid → g ∈$R^{256}$	Computes the element-wise gate used for adaptive CNN–Transformer fusion, as defined in Equation (7).
Fusion	Fusion rule	f = g ⊙ ĉ + (1−g) ⊙ t˜	Adaptive weighting of CNN-derived and Transformer-derived features, as defined in Equation (8).
Classifier	Hidden layer	FC(256→128) + ReLU+Dropout(0.3)	Matches architecture description.
Classifier	Output layer	FC(128→5) + Softmax	AAMI 5 classes (N, L, R, A, V).
Training	Loss	Weighted categorical cross-entropy	Class weights were computed inversely proportional to class frequency to compensate for heartbeat-category imbalance.
Training	Sampling strategy	Class-balanced sampling	Used during training to improve exposure to less frequent heartbeat categories without altering the validation or testing distributions.
Training	Optimizer	Adam (end-to-end backpropagation)	Updates CNN, Transformer, fusion, classifier jointly.
Training	Learning rate	Not explicitly reported; recommend 1e−3 (tune 1e−4–3e−3)	Report final LR used for reproducibility.
Training	Batch size	Not explicitly reported; recommend 64 (tune 32–256)	Depends on GPU memory and window length.
Training	Epochs/stopping	Not explicitly reported; recommend 50–150 with early stopping (patience 10)	Report criterion (val loss/F1).
Training	Weight decay	Not explicitly reported; recommend 1e−4	Helps generalization under noise.
Training	LR scheduler	Not explicitly reported; recommend cosine decay or step decay	Optional but improves stability.
Training	Random seed	Not explicitly reported; recommend fixed seed (e.g., 42)	Improves reproducibility across runs.

The table lists the main architectural and training configurations used in the proposed CNN–Transformer model with gated fusion. It includes input length, Transformer embedding dimension, positional encoding type, number of Transformer encoder layers, attention heads, feed-forward dimension, dropout, CNN and Transformer feature dimensions, fusion projection layers, gating network structure, classifier layers, loss function, optimizer, and training-related hyperparameters. This table provides implementation-level detail to improve reproducibility and clarify the model configuration used for the reported experiments.

Key configuration settings for the Transformer-based sequence modeling stage in TransECG-Net are summarized in Table 4. TransECG-Net integrates a CNN branch for local morphological feature extraction with a Transformer encoder that models long-range temporal dependencies via multi-head self-attention and positional encoding, followed by a softmax classifier for five-class heartbeat classification under the AAMI-recommended setting (N, L, R, A, V). The supervised model is trained by minimizing cross-entropy loss, and gradient-based optimizers such as Adam are used to update the CNN, Transformer encoder, gated fusion module, and classification layers via backpropagation. For privacy-preserving deployment, a federated learning setting can be enabled where multiple clients train locally and the server aggregates updates using FedAvg across five client nodes under non-IID data distributions, with only minor performance degradation reported (<1.3%).

After defining the model configuration, the evaluation metrics were formulated to quantify both overall classification accuracy and class-wise diagnostic performance.

To provide a comprehensive evaluation beyond overall accuracy, the classification results were analyzed using both global and class-wise metrics. Let *C* denote the five-class confusion matrix, where $C_{ij}$ represents the number of ECG heartbeat samples whose true class is *i* and predicted class is *j*. The five classes were *N*, *L*, *R*, *A*, and *V*, and the total number of testing samples was denoted as $N_{\mathrm{total}}$.

First, the overall classification accuracy was calculated from the diagonal elements of the confusion matrix, as shown in Equation (9):

$$\mathrm{Accuracy}=\frac{\sum_{k=1}^{K}\;C_{kk}}{N_{\mathrm{total}}}$$ (9)

where K=5 is the number of heartbeat classes, $C_{kk}$ is the number of correctly classified samples in class *k*, and $N_{\mathrm{total}}$ is the total number of testing samples. This metric reflects the overall proportion of correctly predicted ECG heartbeat samples.

However, because accuracy alone does not describe false-positive and false-negative behavior for each heartbeat category, class-wise metrics were also calculated using a one-vs.-rest strategy. For each target class *k*, the true-positive component was first defined as shown in Equation (10):

$$TP_{k}=C_{kk}$$ (10)

where $TP_{k}$ is the number of true-positive samples correctly predicted as class *k*. The false-negative component was then calculated using Equation (11):

$$FN_{k}=\underset{\;j\neq k}{\sum }\;C_{kj}$$ (11)

where $FN_{k}$ is the number of samples belonging to class *k* but incorrectly predicted as another class. The false-positive component was calculated using Equation (12):

$$FP_{k}=\underset{i\neq k}{\sum }\;C_{ik}$$ (12)

where $FP_{k}$ is the number of samples not belonging to class *k* but incorrectly predicted as class *k*. The true-negative component was obtained using Equation (13):

$$TN_{k}=N_{\mathrm{total}}-TP_{k}-FN_{k}-FP_{k}$$ (13)

where $TN_{k}$ is the number of samples that neither belonged to class *k* nor were predicted as class *k*. Based on these components, precision was calculated to quantify the reliability of positive predictions for each class, as given in Equation (14):

$$\mathrm{Precisio}n_{k}=\frac{TP_{k}}{TP_{k}+FP_{k}}$$ (14)

where $TP_{k}+FP_{k}$ represents all samples predicted as class *k*. A higher precision indicates fewer false-positive predictions for that class. Next, recall, also referred to as sensitivity, was calculated to quantify the ability of the model to correctly detect samples belonging to each class, as shown in Equation (15):

$$\mathrm{Recal}l_{k}=\mathrm{Sensitivit}y_{k}=\frac{TP_{k}}{TP_{k}+FN_{k}}$$ (15)

where $TP_{k}+FN_{k}$ represents all true samples of class *k*. A higher recall indicates fewer missed samples for that class. Specificity was then calculated to measure how accurately the model rejected non-target classes, as shown in Equation (16):

$$\mathrm{Specificit}y_{k}=\frac{TN_{k}}{TN_{k}+FP_{k}}$$ (16)

where $TN_{k}+FP_{k}$ represents all samples that did not belong to class *k*. A higher specificity indicates stronger suppression of false alarms. Finally, the F1-score was calculated as the harmonic mean of precision and recall, as given in Equation (17):

$$F1_{k}=\frac{2\times \mathrm{Precisio}n_{k}\times \mathrm{Recal}l_{k}}{\mathrm{Precisio}n_{k}+\mathrm{Recal}l_{k}}$$ (17)

where $\mathrm{Precisio}n_{k}\;$ and $\mathrm{Recal}l_{k}$ are the class-wise precision and recall values for class *k*. The F1-score provides a balanced measure of positive predictive reliability and detection sensitivity, especially when evaluating abnormal heartbeat categories.

Macro-averaged metrics were calculated by averaging the class-wise values across all five categories, as shown in Equation (18):

$$\mathrm{Macro}\;\mathrm{average}=\frac{1}{K}\overset{K}{\underset{k=1}{\sum }}\;M_{k}$$ (18)

where $M_{k}$ represents the class-wise value of a given metric, such as precision, recall, specificity, or F1-score, and K=5 is the number of classes.

## 3 Results

The generalization performance of the proposed approach was evaluated using the combined ECG dataset constructed from the MIT-BIH Arrhythmia Database and the PhysioNet 2017 Challenge dataset. All steps of the analytical pipeline—including ECG window extraction, preprocessing (segmentation, denoising, and amplitude normalization), model training, and evaluation—were implemented in a Python-based environment. The dataset was split into training/validation/testing subsets (70%/15%/15%), and the test subset was used to assess performance on previously unseen recordings, reflecting the real-world requirement of generalizing across different subjects and acquisition conditions.

**Figure 3:**
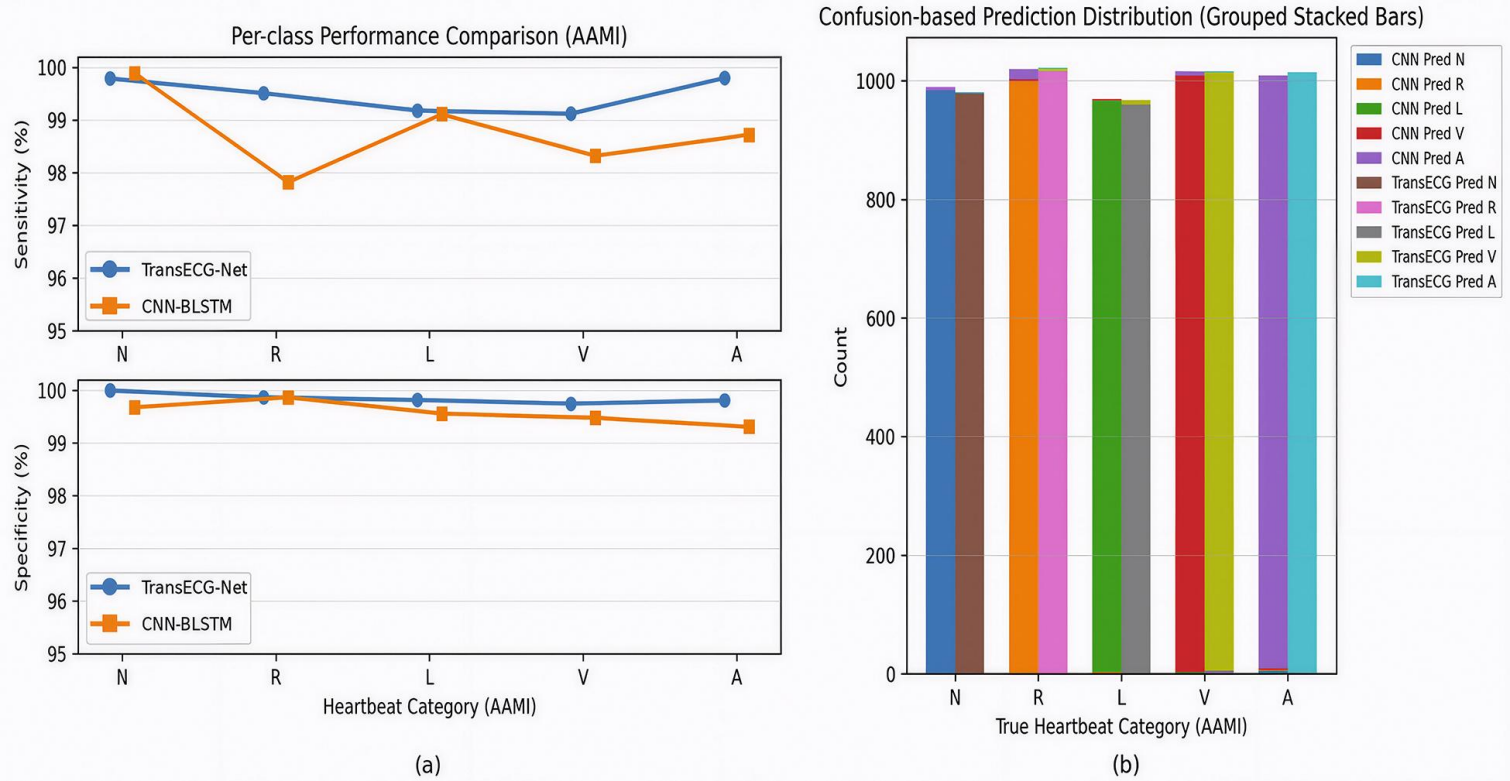
AAMI five-class comparison between TransECG-Net and hybrid CNN-BLSTM. The figure compares class-wise classification behavior between TransECG-Net and the Hybrid CNN-BLSTM baseline under the AAMI-aligned five-class heartbeat setting. Panel-level summaries show sensitivity and specificity across the five categories (N, L, R, A, and V) together with confusion-based prediction distributions. The comparison highlights the improved balance achieved by TransECG-Net between abnormal-beat detection and normal-beat specificity, suggesting that Transformer-based temporal-context modeling provides complementary discriminatory information beyond recurrent sequence modeling.

**Figure 4:**
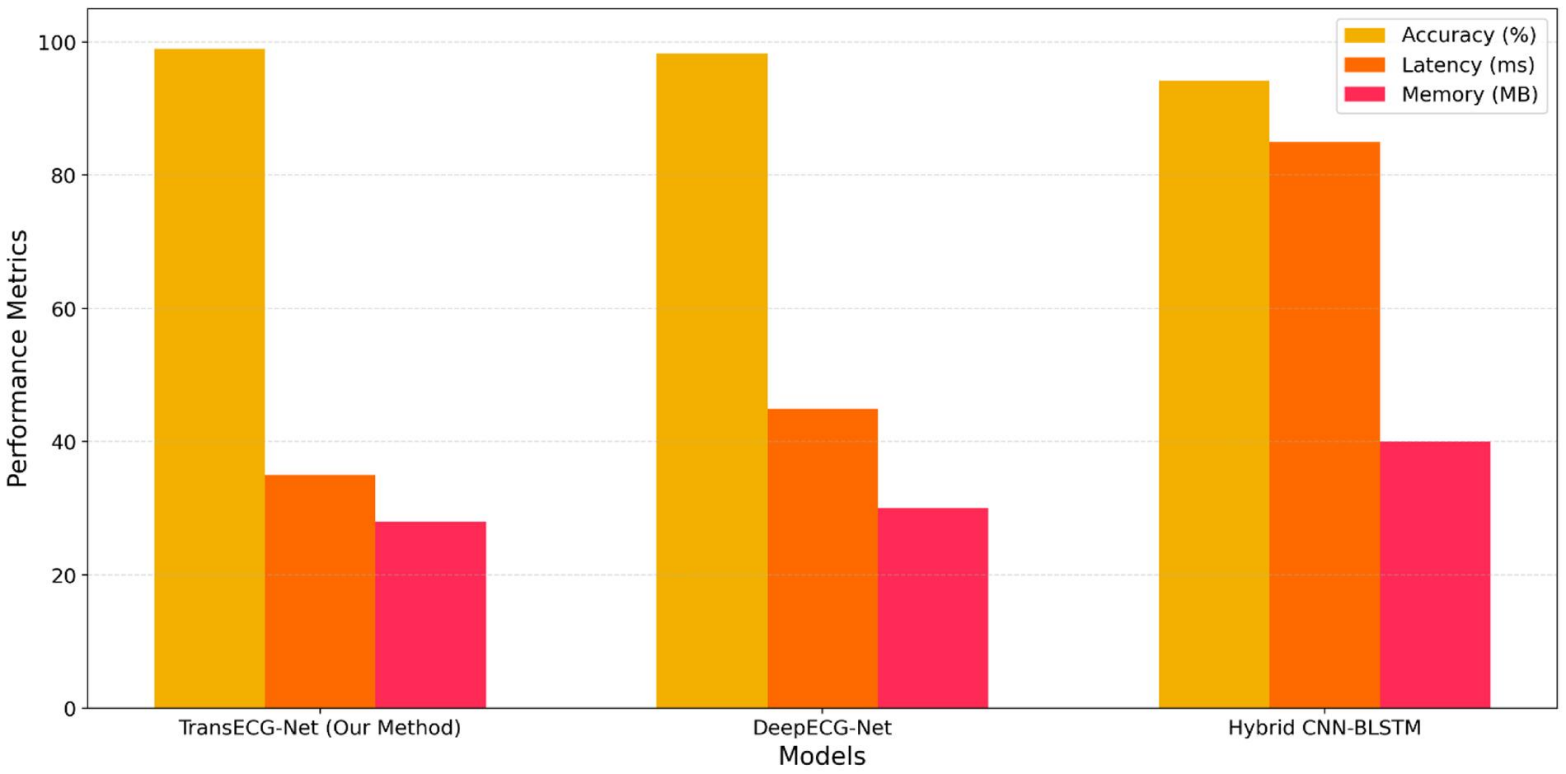
Comparative performance and resource efficiency of TransECG-Net and representative baseline models. The figure summarizes model-level classification performance and deployment-relevant efficiency among TransECG-Net, DeepECG-Net, and Hybrid CNN-BLSTM. Accuracy, inference latency, and memory footprint are compared under the same experimental protocol. TransECG-Net demonstrates the strongest overall trade-off, achieving the highest classification accuracy while maintaining lower latency and smaller memory demand than recurrent or alternative hybrid baselines. These results support the suitability of the proposed CNN–Transformer framework for real-time ECG arrhythmia screening on resource-constrained edge platforms.

To ensure a fair comparison, all competing models were trained and tested under identical data partitions and preprocessing settings. In addition to the proposed TransECG-Net (CNN-Transformer), we evaluated representative baselines including a CNN-only model and recurrent/attention-based sequence models (e.g., CNN-LSTM and CNN-Transformer) as well as ECGNet. Performance was summarized using confusion matrices and standard classification metrics (accuracy, precision, sensitivity/recall, specificity, and F1-score). As reported in Tables 5, 6, TransECG-Net demonstrated improved discrimination across AAMI heartbeat categories compared with the CNN-only baseline. Recurrent and hybrid baseline comparisons are summarized separately in Figures 3, 4 and in the ablation comparison in Table 8. Notably, the proposed model achieved very high specificity for the Normal (N) class, reflecting strong suppression of false alarms-an essential property for scalable screening and continuous monitoring applications.

**Table 5:** Confusion matrix of the CNN-only baseline for AAMI five-class ECG heartbeat classification.

True class	Predicted N	Predicted L	Predicted R	Predicted A	Predicted V	Support
N	983	0	0	6	0	989
L	1	963	2	0	3	969
R	1	0	998	17	3	1,019
A	4	1	2	999	2	1,008
V	0	2	1	8	1,005	1,016
Total predicted	989	966	1,003	1,030	1,013	5,001

The table reports the confusion matrix for the CNN-only baseline, with true heartbeat classes shown as rows and predicted classes shown as columns. The five evaluated categories are N, L, R, A, and V. The CNN-only model correctly classified 4,948 of 5,001 heartbeat samples, corresponding to an overall accuracy of 98.94%. Misclassifications mainly occurred among abnormal classes, indicating that local morphology extraction alone may be insufficient for distinguishing heartbeat categories requiring temporal-context interpretation.

**Table 6:** Confusion matrix of TransECG-Net for AAMI five-class ECG heartbeat classification.

True class	Predicted N	Predicted L	Predicted R	Predicted A	Predicted V	Support
N	979	0	0	2	0	981
L	0	960	0	0	7	967
R	0	0	1,016	2	4	1,022
A	0	1	1	1,012	0	1,014
V	0	3	2	2	1,009	1,016
Total predicted	979	964	1,019	1,018	1,020	5,000

The table reports the confusion matrix for TransECG-Net using the held-out testing set. True classes are displayed as rows and predicted classes as columns for the five AAMI-aligned categories: N, L, R, A, and V. TransECG-Net correctly classified 4,976 of 5,000 heartbeat samples, achieving an overall testing accuracy of 99.52%. Compared with the CNN-only baseline, the reduced cross-class confusion supports the benefit of combining CNN-derived local morphology features with Transformer-derived temporal-context features through gated fusion.

The CNN-only baseline correctly classified 4,948 of 5,001 heartbeat samples, corresponding to an overall accuracy of 98.94%. Most errors occurred between abnormal categories, particularly atrial premature beats and right bundle branch block or ventricular premature beat classes, indicating that local morphology alone may be insufficient for some temporally dependent beat patterns.

TransECG-Net correctly classified 4,976 of 5,000 heartbeat samples, corresponding to an overall accuracy of 99.52%. Compared with the CNN-only baseline, the proposed model reduced cross-class confusion among abnormal heartbeat categories, supporting the benefit of combining local morphology extraction with Transformer-based temporal-context modeling.

To address the limitation of accuracy-only evaluation, additional class-wise metrics were calculated from the TransECG-Net testing confusion matrix using a one-vs.-rest strategy. Precision, recall/sensitivity, specificity, and F1-score were computed for each AAMI heartbeat category to assess both false-positive and false-negative behavior. The resulting class-wise performance profile is summarized in Table 7.

**Table 7:** Class-wise precision, recall, specificity, and F1-score of TransECG-Net for AAMI five-class ECG heartbeat classification.

Class	Support, *n*	TP	FP	FN	Precision (%)	Sensitivity (%)	Specificity (%)	F1-score (%)
N	981	979	0	2	100.00	99.80	100.00	99.90
L	967	960	4	7	99.59	99.28	99.90	99.43
R	1,022	1,016	3	6	99.71	99.41	99.92	99.56
A	1,014	1,012	6	2	99.41	99.80	99.85	99.61
V	1,016	1,009	11	7	98.92	99.31	99.72	99.12
Macro average	—	—	—	—	99.52	99.52	99.88	99.52
Overall accuracy	5,000	4,976	—	—	—	—	—	99.52

The table provides a one-vs-rest class-wise performance analysis derived from the TransECG-Net testing confusion matrix. For each heartbeat category, support, true positives, false positives, false negatives, precision, sensitivity/recall, specificity, and F1-score are reported. TransECG-Net achieved F1-scores of 99.90% for N, 99.43% for L, 99.56% for R, 99.61% for A, and 99.12% for V, with a macro-averaged F1-score of 99.52%. These results confirm that the model performance is not driven by accuracy alone but remains balanced across clinically relevant heartbeat categories.

Based on the TransECG-Net testing confusion matrix, the model correctly classified 4,976 of 5,000 ECG heartbeat samples, corresponding to an overall accuracy of 99.52%. Class-wise analysis showed consistently high performance across all five categories. The F1-scores were 99.90% for N, 99.43% for L, 99.56% for R, 99.61% for A, and 99.12% for V. The macro-averaged precision, recall, specificity, and F1-score were 99.52%, 99.52%, 99.88%, and 99.52%, respectively. These findings indicate that the proposed model was not only accurate overall but also maintained a strong precision–recall balance across normal beats, bundle branch block beats, atrial premature beats, and ventricular premature beats.

### 3.1 Model comparison with representative baselines

Figure 3 summarizes the class-wise comparison between TransECG-Net and the Hybrid CNN-BLSTM baseline, whereas Table 5 provides the confusion matrix for the CNN-only baseline. As reported in Table 5, the CNN-only baseline showed strong performance for the Normal class, although cross-class errors remained among several abnormal heartbeat categories.

To contextualize TransECG-Net from both predictive performance and deployability perspectives, we compare it with two representative hybrid baselines: DeepECG-Net (CNN–Transformer) and Hybrid CNN-BLSTM (CNN–RNN family). As shown in Figure 4, TransECG-Net achieves the best overall trade-off, delivering 99.52% accuracy with 35 ms inference latency and a 28 MB memory footprint. DeepECG-Net attains competitive accuracy (98.3%) but with higher latency (45 ms) and slightly larger memory usage (30 MB). In contrast, Hybrid CNN-BLSTM exhibits notably lower accuracy (94.2%) and substantially higher latency (85 ms) and memory demand (40 MB), consistent with the computational overhead of recurrent processing. Collectively, these results indicate that the proposed CNN–Transformer design with gated fusion sustains state-of-the-art accuracy while remaining well-suited for real-time and edge deployment.

Under the AAMI-recommended 5-class setting, the comparative results consistently show that TransECG-Net delivers both higher accuracy and more stable behavior than representative baselines, including DeepECG-Net (CNN–Transformer) and Hybrid CNN-BLSTM/CNN-LSTM (CNN–RNN family). The per-class statistics and confusion-based distributions further indicate that the proposed model achieves a better balance between abnormal-beat sensitivity and normal-beat specificity, which is essential for screening scenarios where both missed detections and false alarms are costly.

The improvement can be attributed to complementary feature modeling at two granularities. The CNN branch prioritizes local morphological evidence (e.g., P-wave morphology, QRS width, and T-wave polarity), whereas the Transformer branch captures long-range temporal dependencies via self-attention, enabling rhythm-aware discrimination that cannot be reliably inferred from local morphology alone. Crucially, the gated fusion module adaptively reweights morphology-dominant and context-dominant features in a dimension-wise manner, producing a more discriminative representation for clinically similar or challenging categories (e.g., ectopic beats) and reducing cross-class confusion.

Beyond beat-level metrics, TransECG-Net also supports continuous monitoring by summarizing daily outputs into an anomaly rate, computed as the proportion of non-N predictions (L/R/A/V). This aggregation provides an intuitive view of temporal dynamics and helps verify that the model maintains consistent screening behavior under day-to-day variability. Overall, these results support the practicality of the proposed CNN–Transformer framework for both real-time inference and long-term arrhythmia screening, with detailed quantitative comparisons reported in the corresponding tables and figures.

To compare class-wise discrimination against a recurrent hybrid baseline, Figure 3 presents the AAMI five-class sensitivity, specificity, and confusion-based prediction distribution for TransECG-Net and Hybrid CNN-BLSTM.

While Figure 3 focuses on class-wise diagnostic behavior, the next comparison evaluates predictive performance together with practical deployment efficiency. The overall performance–efficiency trade-off among TransECG-Net, DeepECG-Net, and Hybrid CNN-BLSTM is summarized in Figure 4.

Figure 4 compares classification accuracy, inference latency, and memory footprint among TransECG-Net (our method), DeepECG-Net, and Hybrid CNN-BLSTM under the same experimental protocol. The results highlight that TransECG-Net achieves the highest accuracy while maintaining the lowest latency and memory consumption, which is essential for real-time and edge ECG monitoring.

**Figure 5:**
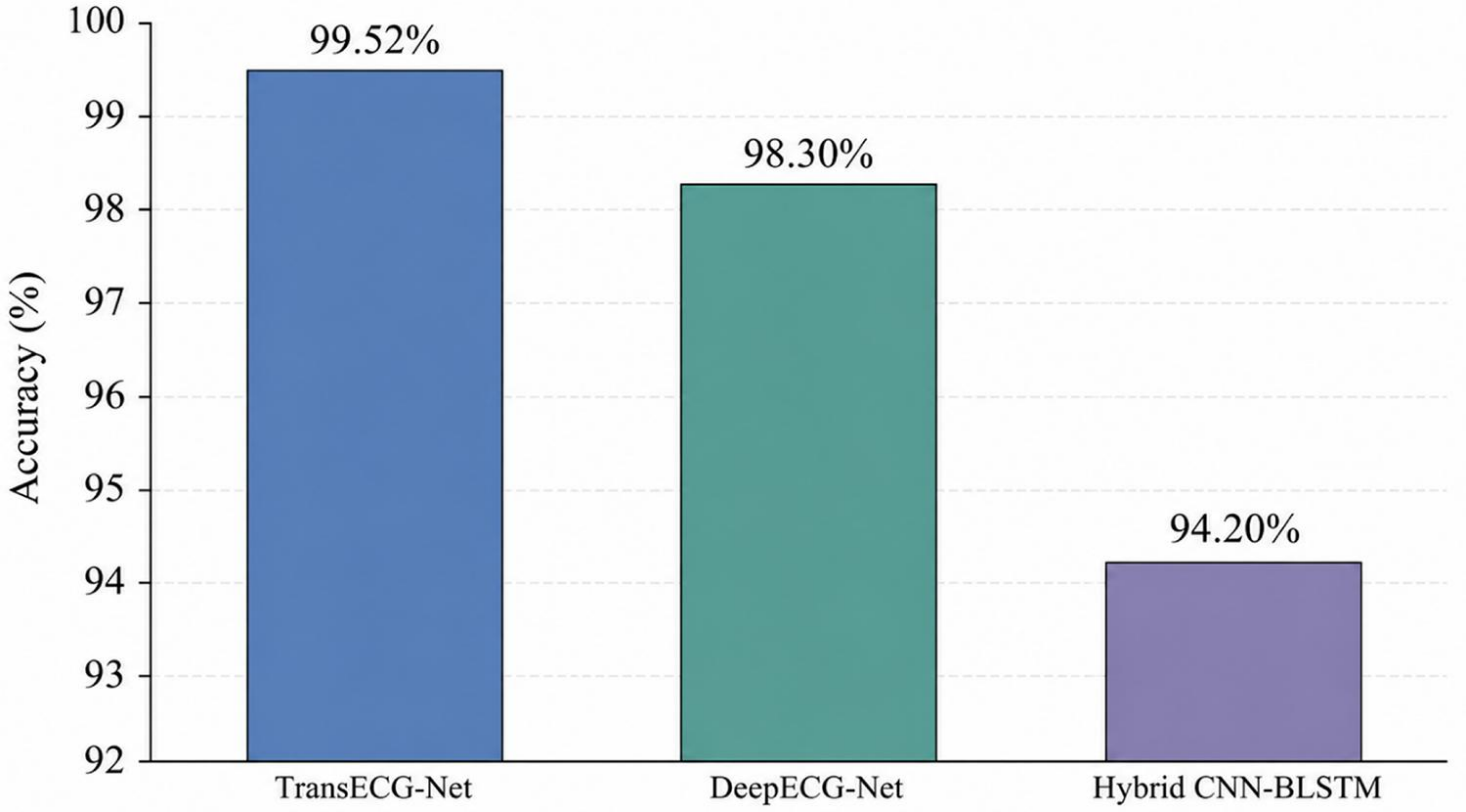
Accuracy-only comparison among TransECG-Net and representative baseline models. The bar plot compares testing accuracy among TransECG-Net, DeepECG-Net, and Hybrid CNN-BLSTM using the same data partition and evaluation protocol. TransECG-Net achieved the highest accuracy (99.52%), followed by DeepECG-Net (98.30%) and Hybrid CNN-BLSTM (94.20%). The accuracy-only view provides a focused visual comparison of the predictive advantage obtained by combining CNN-based morphology extraction, Transformer-based temporal-context modeling, and dimension-wise gated fusion.

To further emphasize the accuracy gains under a zoomed scale, Figure 5 provides an accuracy-only comparison among TransECG-Net (our method), DeepECG-Net, and a Hybrid CNN-BLSTM baseline.

**Figure 6:**
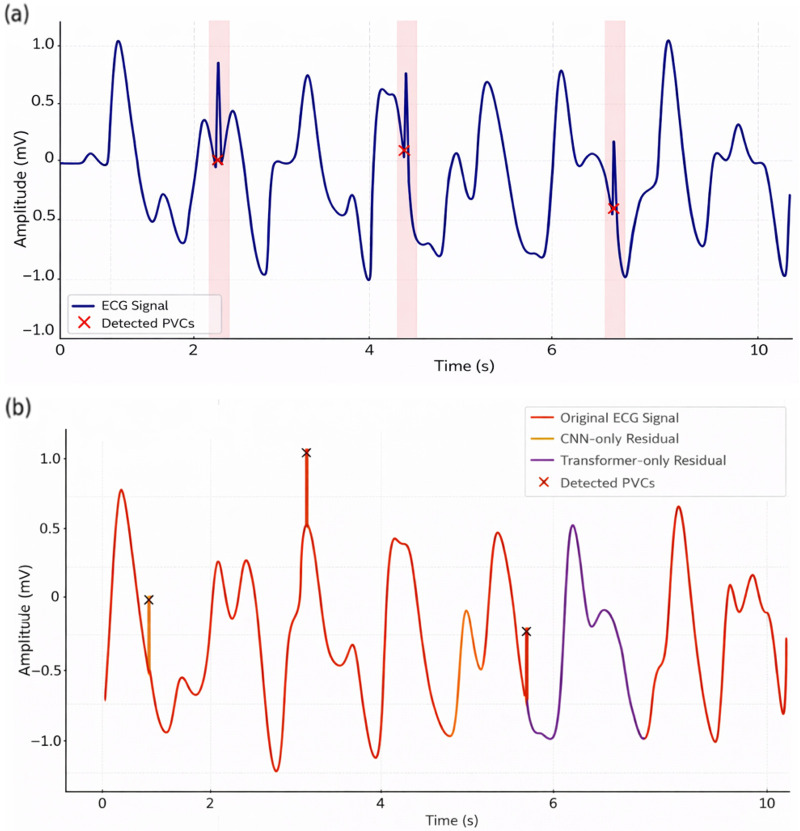
Qualitative visualization of triage-oriented ECG screening and branch-level anomaly response. **(a)** Representative ECG segment with suspected premature ventricular contraction events highlighted to illustrate event localization during screening. **(b)** Residual anomaly-score comparison between CNN-only and Transformer-only branches. The CNN branch emphasizes local morphology changes, whereas the Transformer branch captures broader temporal-context deviations across the ECG window. The complementary response profiles support the use of dimension-wise gated fusion in TransECG-Net, allowing morphology-dominant and context-dominant evidence to be adaptively combined before classification.

Representative qualitative outputs of the proposed screening workflow are shown in Figure 6. Figure 6a illustrates an ECG segment in which suspected premature ventricular contraction events are highlighted to demonstrate triage-oriented event localization, consistent with prior ECG-based PVC detection studies (44). Figure 6b compares residual anomaly-score responses from CNN-only and Transformer-only branches, showing that the two branches provide complementary evidence: the CNN branch is more sensitive to local morphology changes, whereas the Transformer branch captures broader temporal-context deviations. These qualitative findings support the use of gated fusion to combine morphology-dominant and context-dominant representations before final classification.

Signal normalization remains an essential step, as it enforces amplitude consistency across windowed ECG segments and reduces bias introduced by inter-record variability and motion artifacts, thereby enabling reliable comparison and more stable feature learning during end-to-end training.

Traditional machine learning approaches to ECG analysis typically rely on a multi-stage pipeline in which feature engineering and classification are separated, making performance sensitive to handcrafted feature completeness and preprocessing choices. In contrast, TransECG-Net adopts an end-to-end strategy that jointly learns representations and decision boundaries directly from standardized ECG windows. Specifically, the method first applies CNN-based feature extraction to capture local morphological patterns (e.g., PQRST-related structures), and then employs a Transformer encoder with multi-head self-attention and positional encoding to model long-range temporal dependencies across the ECG window without recurrent gating. This hybrid design supports robust arrhythmia classification under realistic conditions and provides a scalable foundation for continuous monitoring and screening applications.

## 4 Discussion

A series of ablation studies was carried out to quantitatively assess the individual impacts of the proposed TransECG-Net hybrid design and its key modeling components. The evaluation framework considered two primary dimensions: (i) diagnostic performance comparison across representative network designs—TransECG-Net (CNN–Transformer), CNN-only, and recurrent baselines such as CNN–Transformer and CNN–LSTM (and/or ECGNet as an established baseline); and (ii) differential performance between the attention-based temporal modeling strategy and alternative temporal modeling settings (e.g., replacing self-attention with recurrent gating, or removing the Transformer stage and using CNN-only). As shown in Figure 4 and further supported by the architecture–activation comparison in Table 8, the TransECG-Net CNN–Transformer configuration achieved the strongest overall performance among the evaluated model variants and representative baselines. The combination of convolutional layers for local morphological pattern extraction with Transformer-based global dependency modeling notably enhanced both sensitivity and specificity while simultaneously lowering false-positive and false-negative rates, which is particularly important in screening-oriented five heartbeat categories ECG detection.

**Table 8:** Quantitative comparison of CNN–transformer and CNN–LSTM variants under different activation functions.

Model	Activation function	Classification performance (%)			
		Acc	Sen	Spe	PPV
CNN-Transformer	mish	99.52 ± 0.08	99.48	99.84	97.56
	ReLU	99.24 ± 0.09	97.89	99.36	96.82
CNN-LSTM	mish	99.01 ± 0.08	97.73	99.44	95.39
	ReLU	98.83 ± 0.09	96.14	98.92	94.45

The table compares CNN–Transformer and CNN–LSTM variants using Mish and ReLU activation functions. Performance is summarized using accuracy, sensitivity, specificity, and positive predictive value. The CNN–Transformer model with Mish activation achieved the strongest overall performance, with an accuracy of 99.52 ± 0.08%, sensitivity of 99.48%, specificity of 99.84%, and PPV of 97.56%. These results support the final architecture choice and indicate that Transformer-based temporal-context modeling provides stronger classification performance than the recurrent CNN–LSTM alternative under the same evaluation setting.

Furthermore, consistent empirical evidence confirmed the advantage of the Transformer-based temporal modeling mechanism over purely convolutional or recurrent alternatives in this screening setting. This improvement can be attributed to self-attention's ability to capture long-range relationships across ECG windows and to emphasize diagnostically informative regions while suppressing less relevant or noise-dominant segments, thereby improving stability and generalization under heterogeneous recording conditions. In addition, when tested under realistic noise perturbations (e.g., baseline wander and white noise with varying SNR), the proposed design maintained a more favorable performance profile than the baselines, indicating stronger robustness for wearable and ambulatory ECG monitoring scenarios.

### 4.1 Autoencoder-based representation learning and denoising

Beyond end-to-end discriminative modeling, autoencoder (AE) families provide an alternative perspective for ECG representation learning, especially under noise-contaminated or weakly labeled settings. In particular, denoising autoencoders (DAE) and convolutional autoencoders can be trained to reconstruct clean ECG from corrupted inputs, thereby learning compact latent representations that preserve morphology while suppressing high-frequency artifacts and baseline wander. Such unsupervised or self-supervised pretraining can be beneficial when annotated arrhythmia labels are limited, as the encoder learns generalizable features that may transfer to downstream classification. Compared with AE-only pipelines, our TransECG-Net directly optimizes the screening objective through supervised learning, while implicitly achieving denoising robustness via the CNN front-end and attention-based context modeling. Nevertheless, AE modules remain attractive as plug-in components: (i) an AE pretraining stage can initialize the CNN encoder for faster convergence and improved stability; (ii) a lightweight DAE front-end can be used as an explicit noise-removal block before the Transformer, potentially improving sensitivity to subtle abnormal beats under real-world noise; and (iii) latent-space regularization may reduce overfitting and improve cross-database generalization. Future work will explore hybrid schemes that combine reconstruction-based self-supervision with classification loss (multi-task learning), enabling the model to simultaneously enhance waveform fidelity and optimize arrhythmia discrimination in long-term monitoring scenarios.

Beyond ECG, hybrid physics-guided/data-driven monitoring and attention-based latent-representation learning have also been explored in complex prognostic and sensor-signal applications (45, 46). Although these studies address non-cardiac systems, they reinforce the broader methodological value of combining domain-guided representation learning with data-driven models, which is consistent with the design rationale of TransECG-Net.

**Figure 7:**
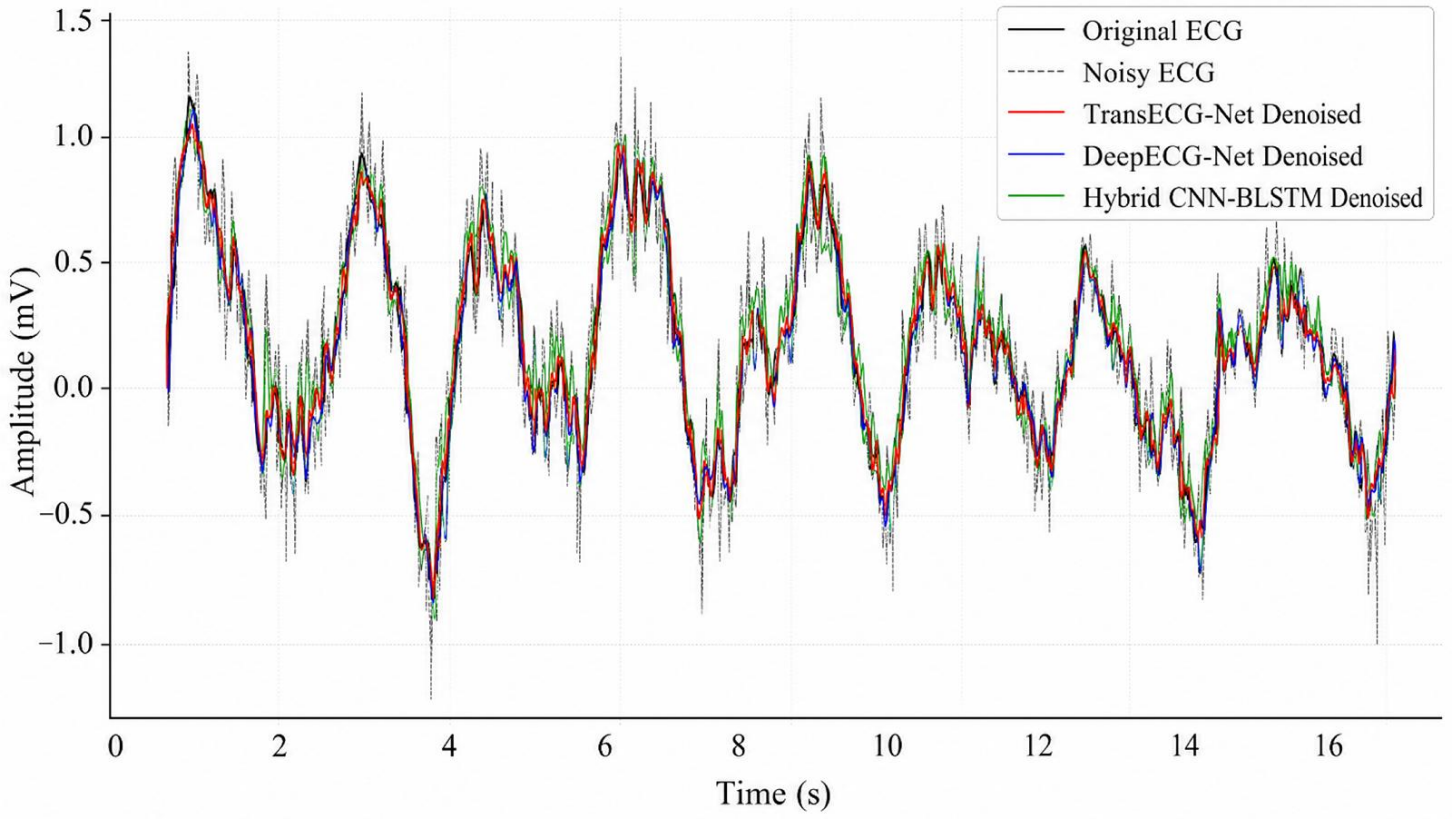
Qualitative comparison of noise robustness via ECG denoising and waveform recovery. Representative ECG waveform recovery results are shown under the same noisy input condition to illustrate robustness against common ambulatory disturbances such as baseline wander and white noise. Compared with baseline methods, TransECG-Net better preserves diagnostically relevant waveform morphology, including QRS-complex structure, local amplitude transitions, and rhythm-related shape patterns. The qualitative comparison supports the model's ability to maintain stable feature extraction under noise-contaminated ECG monitoring conditions.

To provide an intuitive illustration of noise robustness, Figure 7 presents a qualitative comparison of denoised ECG waveforms under the same noisy condition. TransECG-Net more closely follows the original ECG morphology (e.g., around QRS complexes and local amplitude transitions), indicating stable feature extraction and improved tolerance to noise corruption.

**Figure 8:**
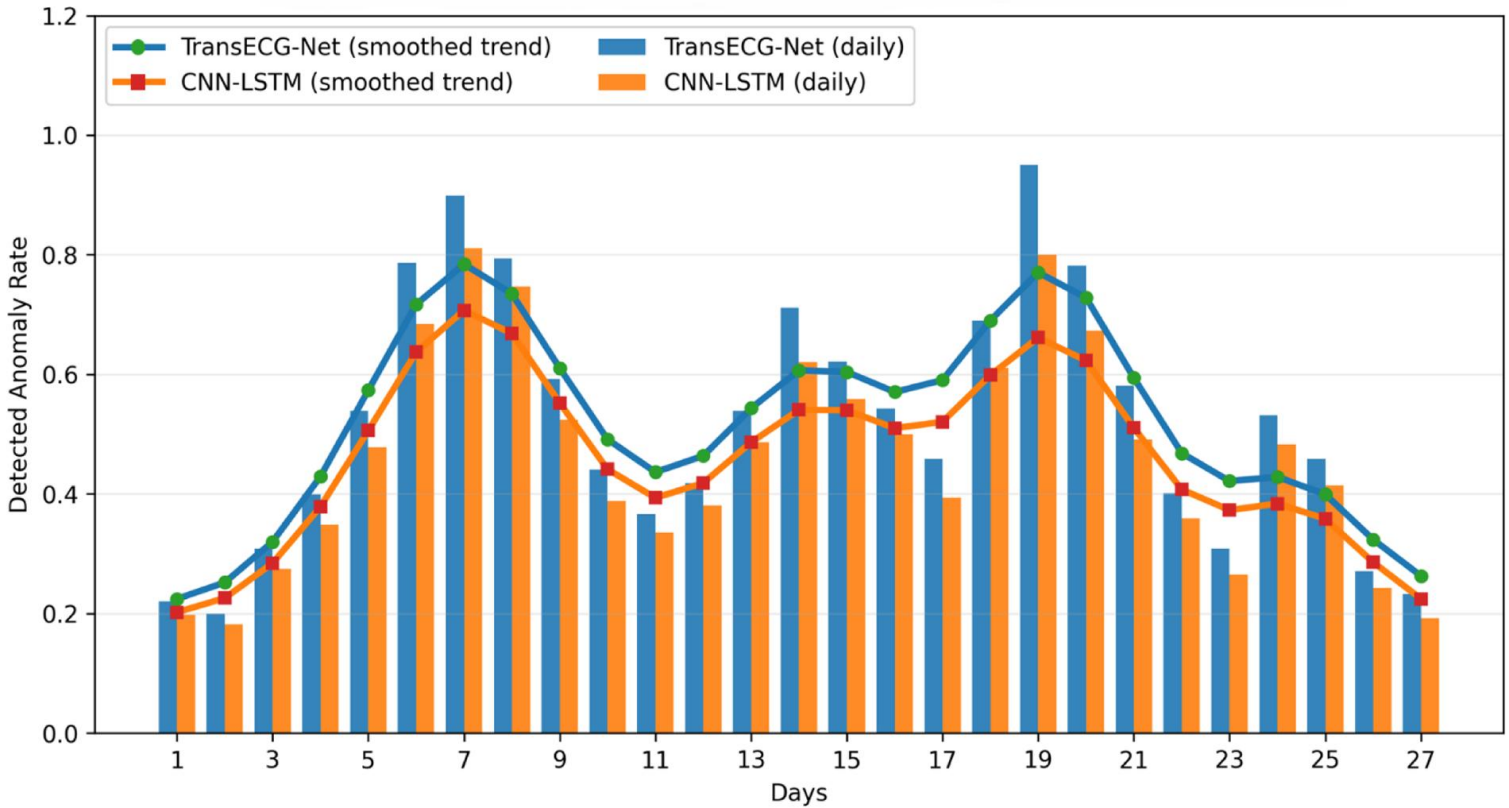
Long-term monitoring example based on daily anomaly-rate aggregation. The figure shows a simulated 30-day continuous monitoring example in which daily anomaly rate is calculated as the proportion of non-normal predictions, with L, R, A, and V treated as abnormal categories relative to N. The daily trend illustrates how beat-level predictions can be aggregated into a clinically interpretable surveillance indicator for downstream alerting and review prioritization. The comparison with the CNN-LSTM baseline demonstrates the potential value of TransECG-Net for stable long-term ECG screening.

Beyond snapshot classification performance, we also evaluate the practical suitability of TransECG-Net for continuous surveillance scenarios. Figure 8 reports the daily anomaly rate over a simulated 30-day monitoring period, computed by aggregating non-N predictions (L/R/A/V) as abnormal. The results illustrate sensitivity to temporal variations in abnormal beat occurrence while maintaining stable trend tracking for downstream alerting.

The application of deep learning-based frameworks to ECG arrhythmia classification offers substantial potential to improve diagnostic throughput while alleviating the workload and cognitive bias of clinicians. The methodology introduced in this study represents a reliable and efficient solution for automated ECG screening that is compatible with real-time deployment constraints. Future investigations may aim to refine the architecture for further edge optimization, expand dataset diversity to strengthen cross-domain generalization, and explore privacy-preserving collaborative training strategies (e.g., federated learning) to support multi-device and multi-site model development without sharing raw patient data.

The spatial features of ECG signals are an indispensable component. Leveraging spatial (morphological) features effectively can enable faster and more efficient ECG signal screening, alleviating the burden on physicians and enhancing work efficiency. Meanwhile, morphological changes in ECG signals are visually observable, and abnormalities caused by variations in waveforms are evident. Observing morphological changes for diagnosing cardiovascular diseases is a common practice among clinicians. Therefore, integrating robust CNN-based spatial feature extraction with effective global temporal dependency modeling is a promising direction for future research.

The hybrid framework combining local feature extraction and temporal dependency learning demonstrates strong potential for biomedical signal processing. Despite advances in ECG intelligence, many existing studies still focus primarily on single-model refinements, which may restrict methodological innovation. By combining a CNN with a Transformer encoder, the proposed method leverages the strengths of both components: CNNs are highly effective at extracting local spatial and morphological features from ECG waveforms, while Transformer encoders excel at capturing long-range temporal dependencies through self-attention without the sequential bottleneck of recurrent gating. This integration enables the model to capture both morphology-related cues and rhythm-level contextual information, improving the efficiency and reliability of ECG arrhythmia classification. Consequently, employing a CNN–Transformer architecture offers a practical pathway for advancing ECG-based screening systems in real-world clinical and wearable deployments, while recurrent architectures (e.g., CNN–Transformer/CNN–LSTM) remain valuable baselines for comparison. Following the long-term monitoring analysis, an ablation-style comparison was conducted to evaluate how architecture choice and activation function, including Mish activation, influenced classification performance (47).

**Figure 9:**
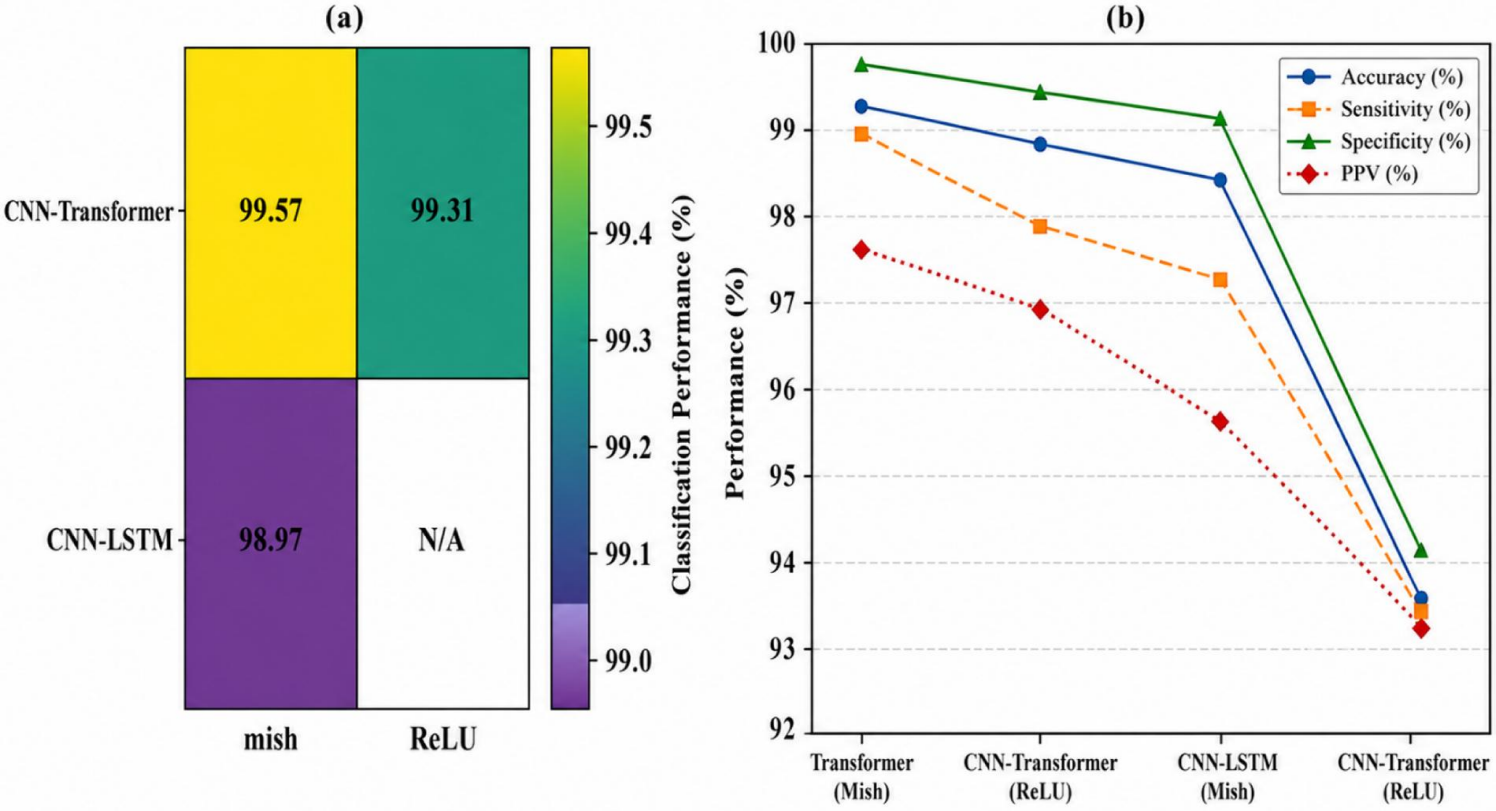
Performance comparison of CNN–transformer and CNN–LSTM variants under different activation functions. The figure summarizes the influence of model architecture and activation function on ECG heartbeat classification performance. CNN–Transformer and CNN–LSTM variants are compared using Mish and ReLU activation functions. Consistent with the quantitative results, the CNN–Transformer configuration with Mish activation achieved the strongest overall performance, supporting the contribution of Transformer-based temporal-context modeling and nonlinear activation selection to the final TransECG-Net design.

As empirically validated in Figure 9, the CNN–Transformer variants outperform the CNN–LSTM variants across key metrics, including sensitivity, specificity, positive predictive value, and accuracy. This performance gap stems from architectural differences: the baseline model mainly captures localized morphological patterns, while the Transformer component extends this to global dependency modeling via multi-head self-attention, enabling modeling of long-range physiological dependencies that are important for arrhythmia recognition. The integrated framework capitalizes on these complementary capabilities-deploying CNN for fine-grained feature extraction and the Transformer for contextual rhythm analysis-yielding a substantial enhancement in classification capability. Consistent with Table 8, the CNN–Transformer model with Mish activation achieved the highest accuracy, sensitivity, specificity, and positive predictive value among the compared architecture–activation variants.

Figure 9 summarizes the effect of model architecture and activation function on ECG heartbeat classification performance. Consistent with Table 8, the CNN–Transformer configuration with Mish activation achieved the highest classification accuracy, sensitivity, specificity, and positive predictive value. Compared with CNN–LSTM variants, the CNN–Transformer models showed stronger overall performance, suggesting that Transformer-based temporal-context modeling provides a more effective complement to CNN-derived morphology features than recurrent sequence modeling in this setting. These findings support the final TransECG-Net configuration used for five-class ECG heartbeat classification.

Evaluation on the public ECG datasets confirms the capability of our model to learn discriminative features directly from windowed ECG signals, leading to strong heartbeat classification performance under the AAMI-recommended setting. In addition to accuracy, precision, and recall, the study emphasizes practical deployability by reporting edge-device results on a Raspberry Pi platform, including sub-50 ms latency and high throughput, supporting real-time screening use cases. A recognized limitation is dataset imbalance and heterogeneity across subjects and acquisition conditions, which can influence generalizability and reduce sensitivity to rare arrhythmia categories. Although the present study used stratified splitting, class-balanced sampling, and weighted cross-entropy loss to reduce imbalance-related bias during training, further improvement may be achieved through additional algorithmic strategies. Future work will therefore prioritize dataset expansion and rebalancing, stronger validation under diverse noise conditions, and the incorporation of imbalance-aware learning methods such as Focal Loss, class-balanced loss functions, minority-aware weighted sampling, and targeted data augmentation to improve recognition of rare or noisy abnormal heartbeat patterns (48, 49).

Ablation studies substantiate that the synergy between the CNN feature extractor and the Transformer encoder is crucial for performance, as they play complementary roles in capturing both localized morphological details and long-range contextual dependencies across the ECG window. This hybrid approach yields a more nuanced interpretation of cardiac dynamics than single-modality networks. Concurrently, the attention mechanism contributes to robustness under noisy monitoring conditions by emphasizing high-relevance temporal segments and suppressing noise-dominant regions, helping maintain strong performance even when noise is injected across SNR levels (0–20 dB). The combined effect of this architectural choice and attention-based robustness facilitates reliable identification of complex pathological patterns and supports practical real-time deployment; moreover, the framework can be extended to privacy-preserving settings using federated learning with minimal performance degradation under non-IID client distributions.

## 5 Conclusions

The analysis of electrocardiogram (ECG) signals is inherently challenging due to their characteristic low frequency components, non-stationary behavior, and vulnerability to diverse real-world noise sources (e.g., motion artifacts and baseline wander). These factors collectively impede efficient and accurate derivation of meaningful high-level representations, particularly in continuous monitoring scenarios where signal quality is highly variable. Conventional machine learning techniques typically depend on manually designed feature extractors crafted from domain expertise. However, such approaches can be limited by feature incompleteness and insufficient nonlinear modeling capacity, which restrict their ability to capture complex and discriminative patterns in ECG data. Consequently, informative structures may be weakened during denoising or feature selection, and downstream classifier performance can become inconsistent across subjects, devices, and recording environments, ultimately compromising screening reliability.

To address these issues, we propose TransECG-Net, a hybrid deep learning architecture that synergistically combines Convolutional Neural Networks (CNNs) with a Transformer encoder. This integrated framework supports end-to-end learning by unifying automated feature extraction and classification, thereby eliminating reliance on manual feature engineering. The CNN module learns compact local morphological representations from fixed-length ECG windows, while the Transformer encoder models long-range dependencies through multi-head self-attention with positional encoding, enabling robust five-class heartbeat classification under the AAMI-recommended setting (N, L, R, A, V). In addition, the framework is evaluated under controlled noise perturbations (SNR stress testing) and validated for edge feasibility, demonstrating suitability for real-time deployment in resource-constrained environments. For continuous monitoring, non-N predictions can be aggregated to compute an anomaly rate for downstream triage. Moreover, the approach can be extended to privacy-preserving training via federated learning, enabling collaborative model improvement without sharing raw patient data.

A key advantage over traditional pipelines is that performance optimization in our model is achieved primarily through architectural design and parameter tuning rather than the laborious process of re-engineering handcrafted features or retraining entirely new classifiers. This results in a more adaptive, noise-robust, and deployment-ready workflow for ECG arrhythmia screening, providing a scalable foundation for clinically viable AI-assisted monitoring systems in both hospital and wearable settings.

## Data Availability

The datasets analyzed in this study are publicly available from PhysioNet, including the MIT-BIH Arrhythmia Database and the PhysioNet/CinC Challenge 2017 dataset. Further inquiries may be directed to the corresponding author.
